# Development of New Benzylpiperazine Derivatives as σ_1_ Receptor Ligands with *in Vivo* Antinociceptive and Anti-Allodynic Effects

**DOI:** 10.1021/acschemneuro.1c00106

**Published:** 2021-05-21

**Authors:** Giuseppe Romeo, Federica Bonanno, Lisa L. Wilson, Emanuela Arena, Maria N. Modica, Valeria Pittalà, Loredana Salerno, Orazio Prezzavento, Jay P. McLaughlin, Sebastiano Intagliata

**Affiliations:** † Department of Drug and Health Sciences, University of Catania, viale A. Doria 6, 95125 Catania, Italy; ‡ Department of Pharmacodynamics, College of Pharmacy, University of Florida, Gainesville, Florida 32610, United States

**Keywords:** Benzylpiperazines, σ receptors, σ-1 antagonist, analgesia, neuropathic pain

## Abstract

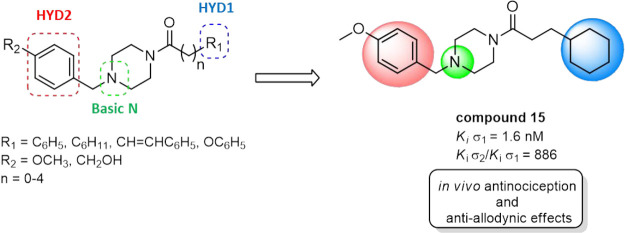

σ-1 receptors (σ_1_R) modulate nociceptive signaling, driving the search for selective antagonists to take advantage of this promising target to treat pain. In this study, a new series of benzylpiperazinyl derivatives has been designed, synthesized, and characterized for their affinities toward σ_1_R and selectivity over the σ-2 receptor (σ_2_R). Notably, 3-cyclohexyl-1-{4-[(4-methoxyphenyl)methyl]piperazin-1-yl}propan-1-one (**15**) showed the highest σ_1_R receptor affinity (*K*_i_ σ_1_ = 1.6 nM) among the series with a significant improvement of the σ_1_R selectivity (*K*_i_ σ_2_/*K*_i_ σ_1_*=* 886) compared to the lead compound **8** (*K*_i_ σ_2_/*K*_i_ σ_1_*=* 432). Compound **15** was further tested in a mouse formalin assay of inflammatory pain and chronic nerve constriction injury (CCI) of neuropathic pain, where it produced dose-dependent (3–60 mg/kg, i.p.) antinociception and anti-allodynic effects. Moreover, compound **15** demonstrated no significant effects in a rotarod assay, suggesting that this σ_1_R antagonist did not produce sedation or impair locomotor responses. Overall, these results encourage the further development of our benzylpiperazine-based σ_1_R antagonists as potential therapeutics for chronic pain.

## Introduction

The σ-1 receptor (σ_1_R) was initially categorized as an opioid receptor subtype because of the binding with the nonselective benzomorphan alazocine (SKF10,047).^1^ Subsequent studies have proven that naloxone did not possess antagonism at this receptor,^2^ and later molecular cloning and the X-ray crystallographic structure of the human σ_1_R revealed no homology with opioid receptors.^3,4^ Indeed, unlike opioid receptors, which possess the seven-transmembrane domain structure characteristics of G protein-coupled receptors, σ_1_R is a transmembrane protein that is present in numerous oligomeric states.^4^ The protomer contains one transmembrane domain, five α helices, and 10 β strands forming the ligand-binding pocket.^4,5^ Therefore, σ_1_R is now recognized as a unique chaperone protein mostly expressed at the endoplasmatic reticulum and is a highly conserved protein among different species with over 90% identical amino acid sequences.^6^

A second σ receptor subtype (named σ_2_R) was discovered and differentiated from the first subtype on the basis of size, tissue distribution, and ligand affinity.^7,8^ σ_2_R has been even more challenging to define than σ_1_R, and its crystal structure has not yet been reported. Indeed, the protein has just recently been cloned, with a sequence identical to the transmembrane protein 97 (TMEM97), a protein involved in cholesterol homeostasis.^9^ Since then, it is general practice to refer to this protein as σ_2_R/TMEM97. Like σ_1_R, σ_2_R/TMEM97 was initially miscategorized and correlated to the progesterone membrane component 1 (PGRMC1), which was thought to be the σ_2_R binding site.^10^ Finally, further studies clarified that σ_2_R/TMEM97 might still interact with PGRMC1 and LDLR (low-density lipoprotein receptor), forming a ternary complex that mediates LDL internalization and trafficking.^11,12^

Both σ receptor (σR) subtypes are present in several CNS areas and peripheral tissues such as the spleen and liver, as well as overexpressed on different human tumors.^6,13^ σ receptors are known to be expressed in key areas of pain control in the CNS such as the locus coeruleus, periaqueductal gray, and rostroventral medulla.^14,15^ In the CNS, these receptors are also very highly expressed in the dorsal root ganglia of the spinal cord, indicating a key role in the function of peripheral pain pathways.^16,17^ In agreement with their anatomical distribution, σRs modulate a broad range of body functions.^18^ Conversely, dysregulation of the physiological activities of σRs has been observed in several medical conditions, including drug addiction, neuropsychiatric disorders, cancer, and chronic pain.^18−22^ With an improving understanding of σRs, σR ligands have recently attracted increased attention from the scientific community for their potential as new medications to treat unmet medical needs,^23−25^ including the novel coronavirus disease 2019 (COVID-19) as recently reported.^26,27^ Notably, novel σR selective ligands are currently under evaluation in clinical trials as diagnostic agents (i.e., PET radiotracers) and therapeutic efficacy for some of the diseases mentioned above.^28−30^

**Figure 1:**
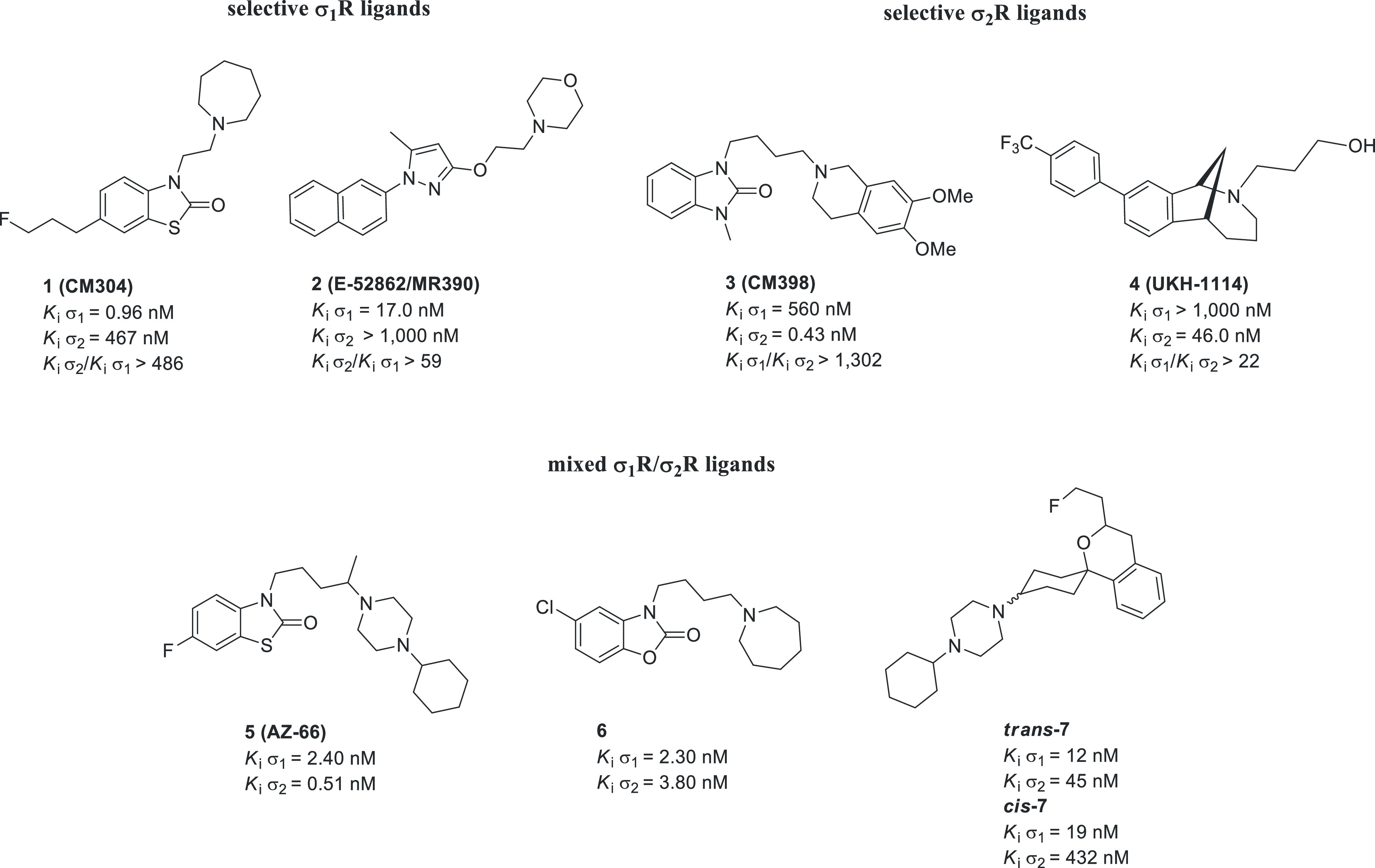
Structures of σRs ligands with antinociceptive activities: selective σ_1_R antagonists **1** and **2**; selective σ_2_R ligands **3** and **4**; mixed σ_1_R/σ_2_R ligands **5**, **6**, *trans***-7**, and *cis***-7**.

Consistent with their molecular role as chaperones, the σRs interact with various proteins, modifying their function.^31,32^ Concerning their active modulatory activity in pain signaling, these protein targets include the μ-opioid receptor, ion channels, and the NMDA receptors.^14^ Significantly, σ_1_R antagonists block the activity of the σ_1_Rs, producing increased opioid and decreased NMDA receptor signaling, thereby enhancing antinociception by opioids and decreasing the hypersensitivity commonly associated with pathological pain.^33^ Interestingly, an increased number of ligands possessing different chemotypes and heterogeneous σRs binding profiles showed a significant antinociception effect in different preclinical *in vivo* pain models (Figure 1).^34−41^

**Figure 2:**
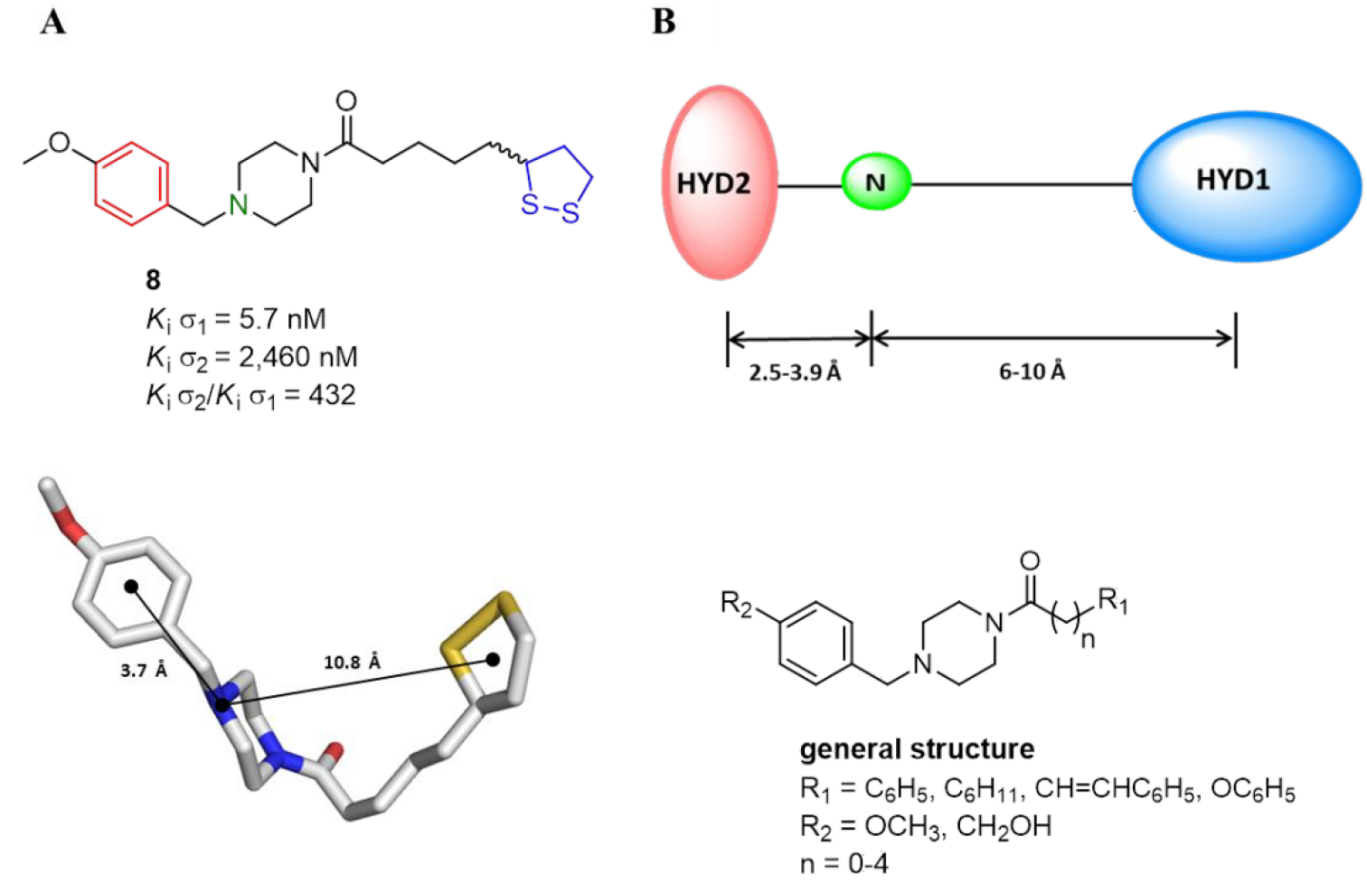
(A) 2D and 3D structures of lead compound **8**. (B) Glennon’s σ_1_R pharmacophoric features (i.e., primary hydrophobic, blue; basic nitrogen, green; secondary hydrophonic, red) and the general structure of newly benzylpiperazine derivatives.

Continuing our efforts to discover selective σ_1_R ligands,^42,43^ in this paper, a set of new benzylpiperazines was synthesized and pharmacologically characterized for their analgesic effects in mice models of pain. Previously, we developed a series of bifunctional σRs ligands with *in vitro* antioxidant properties.^44^ These ligands were obtained by combining a preferred σR cyclic amino moiety, such as benzylpiperazine, with the 1,2-dithiolan-3-yl moiety belonging to the natural antioxidant compound α-lipoic acid (Figure 2A). The 4-methoxybenzylpiperazinyl derivative **8** was previously identified as a potent and selective ligand for the σ_1_R over σ_2_R/TMEM97 (Figure 2A). Moreover, previous structure–affinity relationships (SAfiRs) suggested that the introduction of a para-substituent at the secondary hydrophobic domain (HYD2) improved the affinity and selectivity at σ_1_R.^44^ On the contrary, little exploration of both the chain linker and the primary hydrophobic domain (HYD1) was performed. With this in mind, we used compound **8** as our lead molecule to develop new benzylpiperazine derivatives as potentially more potent and selective σ_1_R ligands over σ_2_R (Figure 2B).

Similar to **8**, the newly synthesized compounds fulfilled Glennon’s pharmacophore model in which two distal hydrophobic regions and a central positive ionizable nitrogen give the essential features for the binding at σ_1_R (Figure 2A,B).^45^ Due to the promising outcomes obtained with previous benzylpiperazines, in this new series, we maintained the 4-methoxybenzylpiperazinyl moiety as the HYD2 and we modified the other distal hydrophobic region and the linker portion (i.e., **13**–**16** and **20**–**22**). Specifically, the substitution of a phenyl, phenoxy, or cyclohexyl group in place of a lipoyl one as the HYD1 was carried out. Additionally, to explore the importance of an additional H-bond donor group for the target binding, the 4-methoxy substituent was replaced with a hydroxymethyl one (**24**).

## Results and Discussion

### Chemistry

**Scheme 1:**
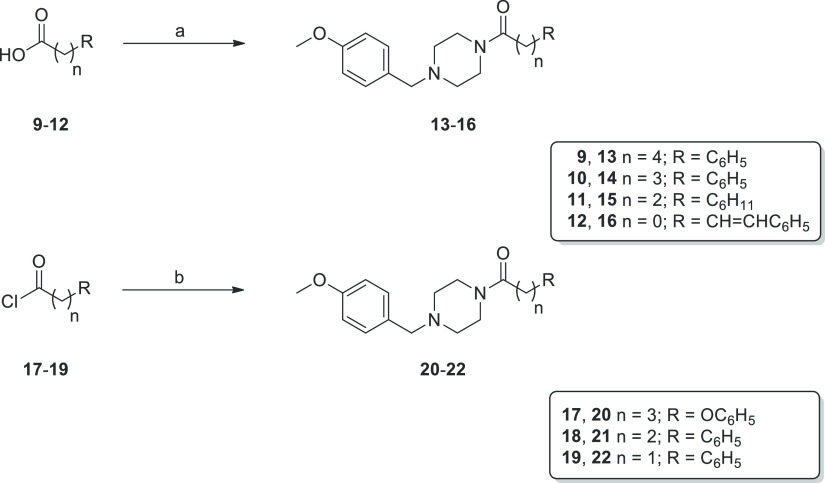
Reaction Pathways for Compounds **13**–**16** and **20**–**22** Reagents and conditions: (a) CDI, dry DCM, RT, then 1-(4-methoxybenzyl)piperazine, 0 °C for 30 min, then RT, 1–2 h, 33–68%; (b) TEA, dry THF, 0 °C for 30 min, then RT, 1–3 days, 61–67%.

**Scheme 2:**
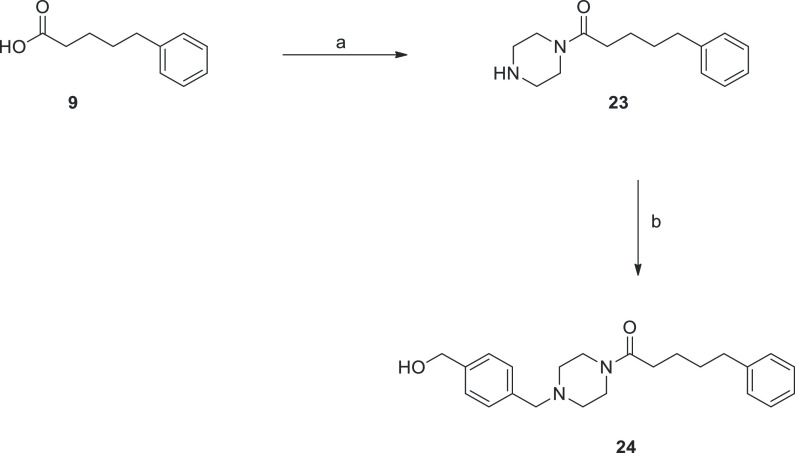
Reaction Pathways for Compounds **23** and **24** Reagents and conditions: (a) CDI, dry DCM, room temperature, then piperazine, 0 °C for 30 min, then RT, 1 h, 75%; (b) 4-(chloromethyl)benzyl alcohol, K_2_CO_3_, KI, DCM, MW (150 W), 120 °C, 2 h, 90%.

Reagents and conditions for preparing the final compounds **13**–**16**, **20**–**22**, and **24** are summarized in Schemes 1 and 2. Precisely, 4-methoxybenzylpiperazinyl analogues **13**–**16** and **20**–**22** were synthesized starting from the suitable activated acids **9**–**12** or acyl chlorides **17**–**19**, according to the two pathways reported in Scheme 1. In the first case, acids **9**–**12** were activated by a reaction with 1,1′-carbonyldiimidazole (CDI) in dry dichloromethane (DCM) to give acyl imidazole intermediates (not isolated), which were then reacted with 1-(4-methoxybenzyl)piperazine in a protective nitrogen atmosphere to afford final amides **13**–**16** in good yields (36–68%). Amides **20**–**22** were obtained directly by the coupling of 1-(4-methoxybenzyl)piperazine with the corresponding organic halides (**17**–**19**) in dry tetrahydrofuran (THF) and using triethylamine as a base catalyst.

The final compound **24** was prepared following the two-step reaction depicted in Scheme 2. The acid derivative **9** was activated by CDI and converted into amide intermediate **23** by reaction with piperazine. Subsequently, treatment with the commercially available 4-(chloromethyl)benzyl alcohol, with K_2_CO_3_ and KI, and using microwaves (MW) irradiation gave the final benzylpiperazine derivative **24** in excellent yield (90%).

Final compounds were characterized as a free base and submitted as such for the *in vitro* binding assay. Compound **15** has been converted into oxalate salt for *in vivo* behavioral studies.

### σR Binding Properties and SAfiRs

The affinities of the newly synthesized benzylpiperazine derivatives (**13**–**16**, **20**–**22**, and **24**) for the σ_1_ and σ_2_ receptors were evaluated in radioligand binding assay using [^3^H]-pentazocine and [^3^H]-DTG, respectively, as radioligands in the presence of haloperidol to determine the nonspecific binding. All tested compounds displayed higher selectivity ratio values (*K*_i_ σ_2_/*K*_i_ σ_1_) than the reference ligand, haloperidol (Table 1). Moreover, compounds **15** and **24** showed improved or similar σ_1_R selectivity (*K*_i_ σ_2_/*K*_i_ σ_1_*=* 886 and 423, respectively) compared to the lead compound **8** (*K*_i_ σ_2_/*K*_i_ σ_1_*=* 432).

**Table 1:** σRs Binding Affinities for **13**–**16**, **20**–**22**, and **24**

	*K*_i_ (nM) ± SD^a^		
compound	σ_1_R	σ_2_R	*K*_i_ σ_2_/*K*_i_ σ_1_
**8**^b^	5.7 ± 0.1	2,460 ± 85	432
**13**	18.1 ± 0.44	1,162 ± 40	64
**14**	13.3 ± 0.22	1,644 ± 37	124
**15**	1.6 ± 0.05	1,418 ± 18	886
**16**	11.3 ± 0.26	3,968 ± 130	351
**20**	102 ± 0.6	4,367 ± 33	43
**21**	8.8 ± 0.22	3,253 ± 40	370
**22**	145 ± 0.5	23,190 ± 146	160
**24**	6.1 ± 0.1	2,583 ± 54	423
haloperidol	1.6 ± 0.1	17.6 ± 0.1	11

a *K*_i_ values are expressed as mean  ±  SD of three independent experiments.

b Data from ref (44).

Regarding the σ_1_R affinity, a clear trend based on the length of the linker chain between the distal phenyl ring and the central amide group can be observed in analogues **13**–**14**, **16**, **21**, and **22** (i.e., ethylene > vinylene ≃ propylene > butylene ≫ methylene). Indeed, chain shortening from four (**13**) to two methylene units (**21**) led to an increase in σ_1_R affinity (*K*_*i*_ σ_1_ = 18.1 and 8.8 nM, respectively); however, a further shortening to only one methylene unit (**22**) was detrimental (*K*_*i*_ σ_1_ = 145 nM). Thus, the length of the ethylene chain in compound **21** produced optimal σ_1_R affinity (*K*_*i*_ = 8.8 nM) and selectivity (370-fold) among this set. A phenyl ring instead of a lipoyl one was tolerated concerning the HYD1 domain, although a loss of selectivity was observed (**8** vs **13**), whereas the introduction of a phenoxy group (**20**) gave the worst result. Further replacement with a cyclohexyl ring resulted in a remarkable improvement of both the affinity and selectivity for σ_1_R (**15** vs **21**).

Finally, an additional H-bond donor group (**24**) in the secondary hydrophobic domain improved neither the σ_1_R affinity nor the selectivity significantly (**24** vs **8**). Compound **15**, which showed the best binding profile among the series (*K*_i_ σ_1_ = 1.6 nM; *K*_i_ σ_2_ = 1,418 nM; *K*_i_ σ_2_/*K*_i_ σ_1_ = 886), was selected for a more in-depth *in vivo* pharmacological evaluation.

### Focally Induced Inflammatory Nociception

**Figure 3:**
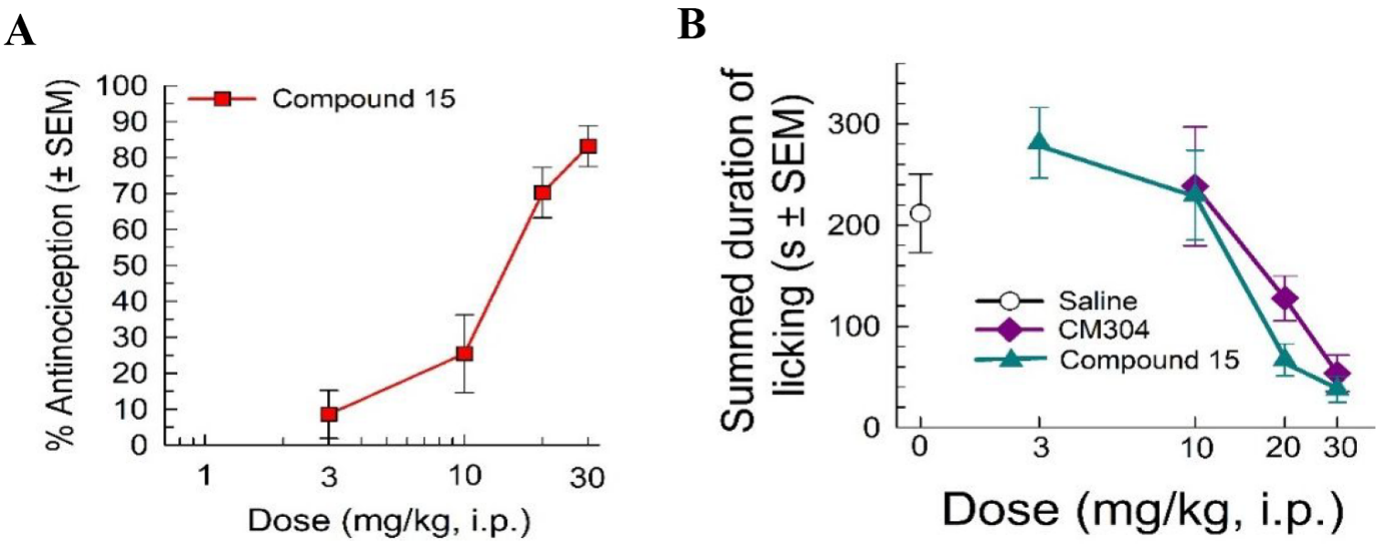
Formalin-induced inflammation assay: (A) compound **15** demonstrates a dose-dependent increase in % antinociception and (B) decreases summed time spent licking of formalin-treated paw in a dose-dependent manner compared to the vehicle control and the selective σ_1_R antagonist (CM304). *n* = 10 for all points.

Due to the lack of reliable *in vitro* assays to establish the agonist/antagonist properties of σRs ligands, the intrinsic functional activity of **15** was assessed by using a behavioral model of nociception. Consistent with the evidence of σ_1_R modulation of nociceptive signaling,^46^ σ_1_R antagonists ameliorate pain responses in a focally induced inflammatory nociception model such as the formalin assay.^15,47,48^ Compound **15** showed significant dose-dependent antinociception in this assay (Figure 3A), consistent with action as a putative σ_1_R antagonist.

Compound **15** showed a similar efficacy at the highest dose in reducing time spent licking the injected paw compared to the positive control CM304 (**1**), a well-characterized selective σ_1_R antagonist (Figure 3B),^35^ with an ED_50_ (and 95% C.I.) value of 12.7 (9.9–16.6) mg/kg, i.p. These results confirm previous reports from Romero et al. in 2012, Gris et al. in 2014, and Cirino et al. in 2019,^35,49,50^ where σ_1_R antagonists blocked peripheral nociception associated with inflammatory pain responses.

### Focally Induced Neuropathy

**Figure 4:**
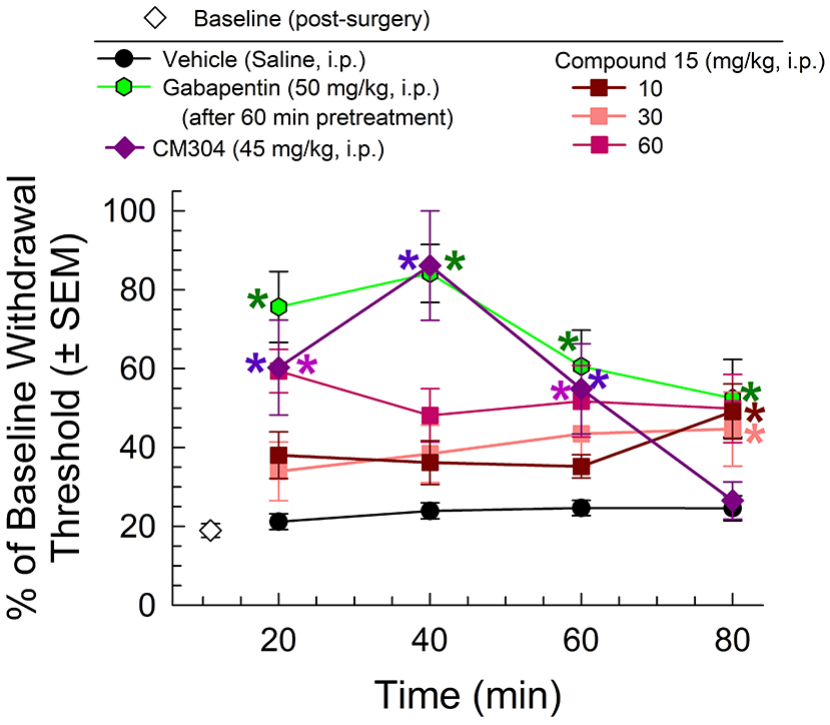
Chronic constriction injury model testing: mechanical allodynia produced from sciatic nerve constriction were reduced after compound **15** treatment, similar to the positive control (gabapentin) with a longer duration of action than the reference compound CM304. *n* = 8–13 for all groups. * = significantly different from vehicle controls; *p* < 0.05.

On the basis of the observed antinociceptive effect exerted by **15** in the formalin assay, we further characterized **15** in a representative model of neuropathic pain. We selected the chronic constriction injury (CCI) model as a widely used and validated assay to produce allodynia.^51−54^ Compound **15** demonstrated significant dose-dependent anti-allodynic effects (*F*_(5, 184)_ = 21.17; *p* < 0.0001; two-way ANOVA with Tukey’s *post hoc* test; Figure 4), with significant increases in withdrawal thresholds at 20, 60, and 80 min post-injection of a 60 mg/kg, i.p. dose (*p* < 0.05; Tukey’s *post hoc* test). These effects were comparable to the results of the positive control gabapentin (Figure 4). CM304, the reference selective σ_1_R antagonist, demonstrated anti-allodynic effects that are comparable to those of gabapentin in a time-dependent manner. The anti-allodynic effects of CM304 peaked at 40 min but began to diminish at 60 min. The current results are consistent with a mechanistic interpretation of anti-allodynia through the σ_1_R antagonism. Notably, CCI produces a focal injury of the sciatic nerve that has been demonstrated to enhance the labeling of spinal σ_1_Rs in a manner enhancing nociceptive signaling.^55^ Currently, these studies with compound **15** verify previous findings stating that noxious stimuli are attenuated by σ_1_R antagonists.^35,48,56^

### Induced Locomotor Activity

**Figure 5:**
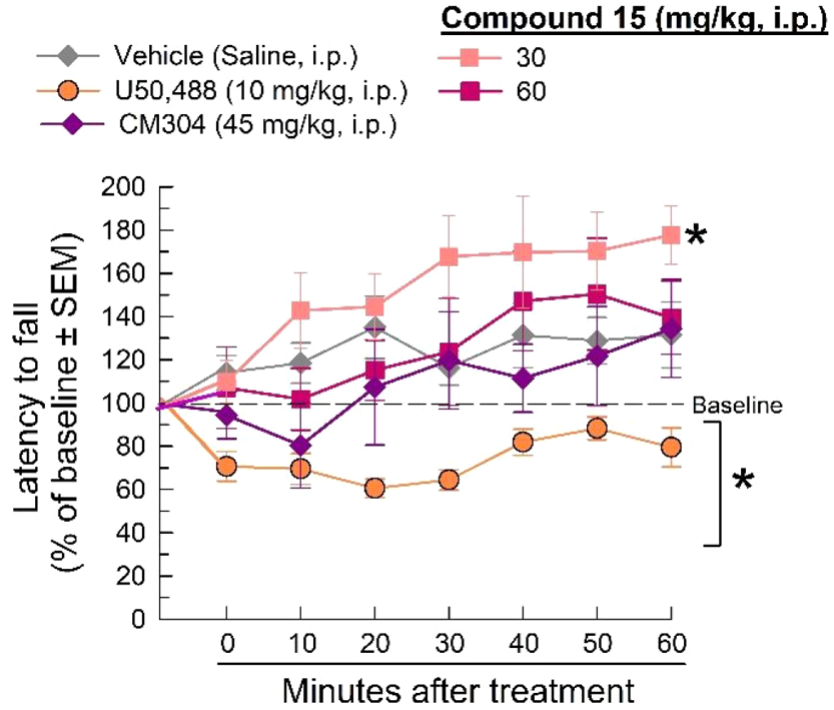
Sedation and evoked, coordinated locomotor function were assessed using the rotarod apparatus following the administration of either saline (i.p.), U50,488 (10 mg/kg, ip), CM304 (45 mg/kg, i.p.), or compound **15** (30 and 60 mg/kg, i.p.). * = significantly different from baseline response (*p* < 0.05). *n* = 8–12.

Treatments for neuropathic pain may be complicated by concordant sedation and impairment of motor function, as demonstrated by gabapentin.^57^ To eliminate the potential complication of impaired locomotion or sedation, the effect of compound **15** on elicited locomotor activity was assessed using the rotarod assay.^35^ The positive control and κ-opioid receptor agonist *trans*-(±)-3,4-dichloro-*N*-methyl-*N*-[2-(1-pyrrolidinyl)cyclohexyl]benzeneacetamide hydrochloride (U50,488, 10 mg/kg, i.p.) significantly impaired evoked locomotor activity compared to the vehicle control (*F*_(4, 301)_ = 26.02; *p* < 0.0001; two-way ANOVA with Tukey’s *post hoc* test; Figure 5) up to 60 min after administration. In contrast, at doses proving effective in the pain assays, compound **15** did not significantly impair evoked locomotor activity, although the 30 mg/kg, i.p. dose produced a singular increase in locomotor performance 60 min post-administration (*p* = 0.003). Although the mechanism of σRs involvement in motor coordination and sedation has not yet been fully defined, the current results confirm the recent finding by Cirino et al.,^35^ showing that selective σ_1_R antagonists fail to produce sedative effects or impair evoked locomotor activity in rodents, confirming their analgesic properties.

## Conclusions

This work has described the design and synthesis of new benzylpiperazinyl derivatives possessing high affinities for σ_1_R (*K*_i_ σ_1_ = 1.6–145 nM) and selectivity over σ_2_R (*K*_i_ σ_2_/*K*_i_ σ_1_ = 43–886). Following Glennon’s structural features criteria necessary for σ_1_R binding, we discovered compound **15** as a potent and selective σ_1_R ligand. Especially, the use of hydrophobic cyclohexyl or phenyl groups and the 4-methoxybenzylpiperazinyl moiety (HYD1 and HYD2, respectively) linked by three-carbon units linker (i.e., **15**, **16**, and **21**) was an excellent combination to obtain optimal σRs binding profiles. Importantly, behavioral pharmacology studies showed that **15** produced significant antinociceptive and anti-allodynic effects in preclinical mouse models of pain without impaired locomotor activity, supporting the development of benzylpiperazine-based σ_1_R antagonists as potential therapeutics for chronic pain.

## Methods

### Chemistry

Melting points were performed in an IA9200 electrothermal apparatus equipped with a digital thermometer in glass capillary tubes and are uncorrected. The elemental analyses for C, H, and N were within ±0.4% of the theoretical values and were recorded on a Carlo Erba elemental analyzer Mod 1108 apparatus. Infrared spectra were determined in KBr disks (solid samples) or NaCl plates (oil samples) on a PerkinElmer 1600 Series FT-IR spectrometer. The ^1^H NMR and ^13^C NMR spectra of intermediate and final compounds were recorded with a Varian Inova Unity (200 MHz) spectrometer and a Varian Inova Unity (500 MHz) spectrometer using a DMSO-*d*_*6*_ solution. The chemical shifts are reported in δ values (ppm), using tetramethylsilane (TMS) as the internal standard; the coupling constants (*J*) are given in hertz (Hz). The signal multiplicities are characterized as s (singlet), d (doublet), t (triplet), or m (multiplet). Microwave irradiation experiments were carried out with a CEM Discovery instrument using closed Pyrex glass tubes with Teflon-coated septa. Thin-layer chromatography (TLC) on Merck plates (aluminum sheet coated with silica gel 60 F_254_) was used to monitor the progress of reactions and to test the purity (≥95%) of all the synthesized compounds, and spots were visualized under UV (λ = 254 and 366 nm) or in an iodine chamber. The purification of synthesized compounds by column chromatography was performed using Merck silica-gel 60 (230–400 mesh). All chemicals and solvents were purchased from commercial vendors and were of reagent grade.

### General Procedure for the Synthesis of 4-(Methoxyphenyl)methylpiperazine Derivatives **13**–**16**

1,1′-Carbonyldiimidazole (1.0 equiv) was mixed to a stirred solution of suitable acid **9**–**12** (1.0 equiv) in dry DCM (6 mL) at room temperature. Then, after no gas evolution was observed, the mixture thus obtained was added dropwise to a stirred solution of 1-(4-methoxybenzyl)piperazine (1.1 equiv) in dry DCM (6 mL) at 0 °C under a nitrogen atmosphere. The reaction was carried out for 30 min at 0 °C and then for 1–2 h at room temperature. The mixture was washed with 10% aqueous NaCl solution (4 × 10 mL) and H_2_O (2 × 10 mL). The organic layer was dried over anhydrous sodium sulfate and then evaporated under reduced pressure to obtain a crude, which was purified as specified for each final product.

#### 1-{4-[(4-Methoxyphenyl)methyl]piperazin-1-yl}-5-phenylpentan-1-one **(13)**

The yellow crude oil was purified by flash column chromatography using ethyl acetate/methanol (9.5:0.5, v/v) as an eluent to afford **13** (0.547 g, 60.4%) as a colorless oil. IR (neat, selected lines): cm^–1^ 3447, 2945, 1646, 1508, 1458, 1242, 748. ^1^H NMR (200 MHz, DMSO-*d*_6_): δ 7.00–7.35 (m, 5H + 2H, aromatic), 6.80–6.95 (m, 2H, aromatic), 3.73 (s, 3H, OCH_3_), 3.35–3.50 (m, 4H + 2H, piperazine + ArC*H*_2_N), 2.57 (t, *J* = 7.0 Hz, 2H, NCOC*H*_2_CH_2_), 2.10–2.40 (m, 4H + 2H, piperazine + CH_2_C*H*_2_C_6_H_5_), 1.35–1.65 (m, 4H, CH_2_C*H*_2_C*H*_2_CH_2_). ^13^C NMR (126 MHz, DMSO-*d*_6_): δ 170.4, 158.3, 142.1, 130.1, 129.6, 128.3, 128.2, 125.6, 113.6, 61.3, 55.0, 52.7, 52.2, 44.9, 41.0, 34.9, 32.1, 30.6, 24.4. Anal. calcd for C_23_H_30_N_2_O_2_: C, 75.37; H, 8.25; N, 7.64. Found: C, 75.15; H, 8.32; N, 7.56.

#### 1-{4-[(4-Methoxyphenyl)methyl]piperazin-1-yl}-4-phenylbutan-1-one (**14**)

The crude was purified by recrystallization from ethanol/water (1:2, v/v) to afford **14** (0.431 g, 54.5%) as white crystals. Mp: 91.0–93.9 °C. IR (KBr, selected lines): cm^–1^ 3028, 2952, 1640, 1509, 1240. ^1^H NMR (200 MHz, DMSO-*d*_6_): δ 7.37–7.10 (m, 5H + 2H, aromatic), 6.95–6.80 (m, 2H, aromatic), 3.72 (s, 3H, OCH_3_), 2.57 (s, 2H, ArCH_2_N), 3.50–3.20 (m, 4H, piperazine), 3.40 (t, *J* = 7.2 Hz, 2H, NCOC*H*_2_CH_2_), 2.40–2.20 (m, 4H + 2H, piperazine + CH_2_C*H*_2_C_6_H_5_), 1.85–1.60 (m, 2H, CH_2_C*H*_2_CH_2_). ^13^C NMR (50 MHz, DMSO-*d*_6_): δ 170.2, 158.4, 141.9, 130.2, 129.6, 128.3, 125.8, 113.6, 61.3, 55.0, 52.8, 52.3, 44.9, 41.0, 34.7, 31.7, 26.8. Anal. calcd for C_22_H_28_N_2_O_2_: C, 74.97; H, 8.01; N, 7.95. Found: C, 75.11; H, 8.22; N, 7.83.

#### 3-Cyclohexyl-1-{4-[(4-methoxyphenyl)methyl]piperazin-1-yl}propan-1-one (**15**)

Compound **15** was prepared by the general procedure described for the synthesis of derivatives **13**–**16** using dry THF instead of dry DCM as a solvent. The yellow crude oil was purified by flash column chromatography using ethyl acetate/methanol (9.7:0.3, v/v) as an eluent to afford **15** (0.360 g, 54.4%) as a white solid. Mp: 69.6–72.5 °C. IR (KBr, selected lines): cm^–1^ 3064, 3029, 2828, 1643, 1277, 1037, 737. ^1^H NMR (free base, 200 MHz, DMSO-*d*_6_): δ 7.25–7.15 (m, 2H, aromatic), 6.95–6.85 (m, 2H, aromatic), 3.73 (s, 3H, OCH_3_), 3.50–3.20 (m, 4H, piperazine), 3.40 (s, 2H, ArCH_2_N), 2.40–2.10 (m, 4H + 2H, piperazine + NCOC*H*_2_CH_2_), 1.75–1.45 (m, 5H, cyclohexane), 1.40–1.00 (m, 2H + 4H, CH_2_C*H*_2_C_6_H_11_ + cyclohexane), 1.00–0.70 (m, 2H, cyclohexane). ^13^C NMR (oxalate salt, 126 MHz, DMSO-*d*_6_): δ 171.1, 163.2, 159.4, 131.7, 125.0, 114.0, 59.7, 55.2, 51.5, 51.1, 43.2, 36.8, 32.7, 32.2, 29.7, 26.2, 25.8. Anal. calcd for C_21_H_32_N_2_O_2_: C, 73.22; H, 9.36; N, 8.13. Found: C, 73.02; H, 9.13; N, 8.32.

#### (2*E*)-1-{4-[(4-Methoxyphenyl)methyl]piperazin-1-yl}-3-phenylprop-2-en-1-one (**16**)

The yellow crude oil was purified by flash column chromatography using ethyl acetate/methanol (9.7:0.3, v/v) as an eluent to afford **16** (0.139 g, 32.8%) as a light yellow solid. Mp: 120.8–122.6 °C. IR (KBr, selected lines): cm^–1^ 2986, 1651, 1605, 1455, 1236. ^1^H NMR (200 MHz, DMSO-*d*_6_): δ 7.80–7.60 (m, 2H, aromatic), 7.55–7.31 (m, 3H + 1H, aromatic + COCH=C*H*), 7.30–7.12 (m, 2H + 1H, aromatic + COC*H*=CH), 6.95–6.80 (m, 2H, aromatic), 3.74 (s, 3H, OCH_3_), 3.80–3.25 (m, 4H, piperazine), 3.43 (s, 2H, ArCH_2_N), 2.55–2.20 (m, 4H, piperazine). ^13^C NMR (126 MHz, DMSO-*d*_6_): δ 164.4, 158.3, 141.5, 135.1, 130.2, 129.6, 129.5, 128.7, 128.0, 118.2, 113.6, 61.2, 55.0, 53.1, 52.2, 45.1, 41.7. Anal. calcd for C_21_H_24_N_2_O_2_: C, 74.97; H, 7.19; N, 8.33. Found: C, 74.78; H, 7.32; N, 8.11.

### General Procedure for the Synthesis of 4-(Methoxyphenyl)methylpiperazine Derivatives **20**–**22**

A mixture of 1-(4-methoxybenzyl)piperazine (1.0 equiv) and triethylamine (1.0 equiv) in dry THF (5 mL) was prepared and left under stirring for 10 min at 0 °C. Subsequently, the appropriate acyl chloride (**17**–**19**, 1.0 equiv) was added to the obtained solution, and the reaction was carried out at 0 °C for 30 min and then at room temperature for 1–3 days. At the end of the reaction time, the solvent was evaporated to dryness under a vacuum. The crude product thus obtained was solubilized in DCM and then washed with a water solution of Na_2_CO_3_ 0.1 M (2 × 20 mL) and NaCl 10% (20 mL). The organic layer was dried over anhydrous sodium sulfate and evaporated under reduced pressure to obtain a residue, which was purified as specified for each final product.

#### 1-{4-[(4-Methoxyphenyl)methyl]piperazin-1-yl}-4-phenoxybutan-1-one (**20**)

The yellow crude oil was triturated with light petroleum ether at 40–60 °C to give a white solid, which was collected, washed with petroleum ether, and dried. The crude thus obtained was purified by recrystallization from ethanol/water (1:2, v/v) to afford **20** (0.277 g, 59.8%) as white crystals. Mp: 88.3–91.3 °C. IR (KBr, selected lines): cm^–1^ 3058, 2945, 1647, 1252, 757. ^1^H NMR (200 MHz, DMSO-*d*_6_): δ 7.35–7.15 (m, 2H + 2H, aromatic), 7.00–6.80 (m, 2H + 3H, aromatic), 3.96 (t, *J* = 6.4 Hz, 2H, CH_2_C*H*_2_OPh), 3.73 (s, 3H, OCH_3_), 3.50–3.20 (m, 4H, piperazine), 3.39 (s, 2H, ArCH_2_N), 2.45 (t, *J* = 7.2 Hz, 2H, NCOC*H*_2_CH_2_), 2.35–2.20 (m, 4H, piperazine), 2.00–1.80 (m, 2H, CH_2_C*H*_2_CH_2_). ^13^C NMR (50 MHz, DMSO-*d*_6_): δ 170.0, 158.5, 158.4, 130.2, 129.6, 129.5, 120.5, 114.4, 113.6, 66.7, 61.3, 55.0, 52.7, 52.3, 44.9, 41.1, 28.6, 24.5. Anal. calcd for C_22_H_28_N_2_O_3_: C, 71.71; H, 7.66; N, 7.60. Found: C, 71.94; H, 7.75; N, 7.73.

#### 1-{4-[(4-Methoxyphenyl)methyl]piperazin-1-yl}-3-phenylpropan-1-one (**21**)

The yellow crude oil was purified by flash column chromatography using ethyl acetate/methanol (9.5:0.5, v/v) as an eluent to afford **21** (0.289 g, 67.9%) as a colorless oil. IR (neat, selected lines): cm^–1^ 3482, 2934, 2806, 1637, 1512, 1245, 1032, 999, 701. ^1^H NMR (200 MHz, DMSO-*d*_6_): δ 7.35–7.10 (m, 5H + 2H, aromatic), 6.95–6.80 (m, 2H, aromatic), 3.73 (s, 3H, OCH_3_), 3.55–3.20 (m, 4H, piperazine), 3.38 (s, 2H, ArCH_2_N), 2.79 (t, *J* = 7.8 Hz, 2H, NCOC*H*_2_CH_2_), 2.58 (t, *J* = 7.8 Hz, 2H, CH_2_C*H*_2_C_6_H_5_), 2.40–2.15 (m, 4H, piperazine). ^13^C NMR (126 MHz, DMSO-*d*_6_): δ 169.8, 158.3, 141.4, 130.1, 129.6, 128.4, 128.2, 125.9, 113.6, 61.3, 55.0, 52.6, 52.2, 44.9, 41.1, 33.9, 30.8. Anal. calcd for C_21_H_26_N_2_O_2_: C, 74.52; H, 7.74; N, 8.28. Found: C, 74.41; H, 7.60; N, 8.10.

#### 1-{4-[(4-Methoxyphenyl)methyl]piperazin-1-yl}-2-phenylethan-1-one (**22**)

The yellow crude oil was purified by flash column chromatography using ethyl acetate/methanol (9.5:0.5, v/v) as an eluent to afford **22** (0.252 g, 61.7%) as a white solid. Mp: 97.6–99.8 °C. IR (KBr, selected lines): cm^–1^ 3032, 3018, 2924, 2802, 1647, 1438, 1235, 1036, 794. ^1^H NMR (200 MHz, DMSO-*d*_6_): δ 7.35–7.10 (m, 5H + 2H, aromatic), 6.93–6.80 (m, 2H, aromatic), 3.73 (s, 3H, OCH_3_), 3.69 (s, 2H, NCOC*H*_2_C_6_H_5_), 3.50–3.30 (m, 4H, piperazine), 3.38 (s, 2H, ArCH_2_N), 2.30–2.15 (m, 4H, piperazine). ^13^C NMR (50 MHz, DMSO-*d*_6_): δ 168.8, 158.4, 135.9, 130.2, 129.6, 129.0, 128.4, 126.4, 113.6, 61.3, 55.0, 52.7, 52.1, 45.5, 41.3. Anal. calcd for C_20_H_24_N_2_O_2_: C, 74.04; H, 7.46; N, 8.64. Found: C, 73.87; H, 7.27; N, 8.55.

### 5-Phenyl-1-(piperazin-1-yl)pentan-1-one (**23**)

1,1′-Carbonyldiimidazole (0.910 g, 5.61 mmol) was added to a solution of 5-phenyl-valeric acid (**9**) (0.80 g, 4.49 mmol) in dry DCM (8 mL) at room temperature. Then, after no gas evolution was observed, the mixture thus obtained was added dropwise to a stirred solution of piperazine (1.93 g, 22.44 mmol) in dry DCM (10 mL) at 0 °C, under a nitrogen atmosphere. The reaction was carried out for 30 min at 0 °C and for 1 h at room temperature. The mixture was washed with 10% aqueous NaCl solution (4 × 10 mL) and H_2_O (2 × 10 mL). The organic layer was dried over anhydrous sodium sulfate and evaporated to dryness under reduced pressure to obtain **23** (0.82 g, 73.8%) as a pure yellow oil and used for the next step without further purification. IR (KBr, selected lines): cm^–1^ 3460, 2936, 1652, 1455, 701. ^1^H NMR (200 MHz, DMSO-*d*_6_): δ 7.34–7.09 (m, 5H, aromatic), 3.60–2.80 (m, 4H, piperazine), 2.68–2.46 (m, 4H + 2H, piperazine + NCOC*H*_2_CH_2_), 2.28 (t, *J* = 7.2 Hz, 2H, CH_2_C*H*_2_C_6_H_5_), 1.68–1.48 (m, 4H, CH_2_C*H*_2_C*H*_2_CH_2_). Anal. calcd for C_15_H_22_N_2_O: C, 73.13; H, 9.00; N, 11.37. Found: C, 73.00; H, 9.15; N, 11.17.

### 1-(4-{[4-(Hydroxymethyl)phenyl]methyl}piperazin-1-yl)-5-phenylpentan-1-one (**24**)

A mixture of compound **16** (0.765 g, 2.98 mmol), K_2_CO_3_ (0.619 g, 4.48 mmol), a catalytic amount of KI, and [4-(chloromethyl)phenyl]methanol (0.146 g, 3.58 mmol) in DCM (2 mL) was placed in a 10 mL Pyrex glass tube, sealed with a Teflon-coated septum. The mixture was heated and stirred at 120 °C under microwave irradiations for 2 h (run time 2 min, microwave max power 150W, max pressure 150 Psi). Subsequently, the reaction mixture was washed with 10% aqueous NaCl solution (4 × 10 mL) and H_2_O (2 × 10 mL). The organic layer was dried over anhydrous sodium sulfate and evaporated to dryness under reduced pressure to obtain a yellow oil. The purification of the crude product was performed by flash column chromatography using ethyl acetate/methanol (9:1, v/v) as an eluent to afford **24** (0.54 g, 91.7%) as a light yellow oil. IR (KBr, selected lines): cm^–1^ 3420, 3024, 2933, 1636, 1458, 1346, 1231, 1000, 701. ^1^H NMR (200 MHz, DMSO-*d*_6_): δ 7.35–7.10 (m, 5H + 4H, aromatic), 5.16 (t, *J* = 5.7 Hz, 1H, CH_2_O*H*), 4.47 (d, *J* = 5.7 Hz, 2H, C*H*_2_OH), 3.50–3.15 (m, 4H, piperazine), 3.44 (s, 2H, ArC*H*_2_N), 2.57 (t, *J* = 7.2 Hz, 2H, NCOC*H*_2_CH_2_), 2.40–2.10 (m, 2H + 4H, CH_2_C*H*_2_C_6_H_5_ + piperazine), 1.70–1.35 (m, 4H, CH_2_C*H*_2_C*H*_2_CH_2_). ^13^C NMR (126 MHz, DMSO-*d*_6_): δ 170.4, 142.1, 141.3, 136.1, 128.7, 128.3, 128.2, 126.4, 125.6, 62.7, 61.7, 52.9, 52.3, 44.9, 41.0, 34.9, 32.1, 30.5, 24.4. Anal. calcd for C_23_H_30_N_2_O_2_: C, 75.37; H, 8.25; N, 7.64. Found: C, 75.20; H, 8.09; N, 7.50.

### σRs Binding Assays

Binding tests were performed following known reported protocols.^39,44^ The binding assay for σ_1_R was carried out using guinea pig brain membrane homogenates according to DeHaven-Hudkins et al.,^58^ while the binding assay for σ_2_R was performed following experimental procedures described by Mach et al.^59^ Inhibitory constants (*K*_i_) were calculated using the radioligand binding analysis software EBDA/Ligand (Elsevier/Biosoft).

### Behavioral Pharmacology

#### Animals

Adult male C57BL/6J and CD-1 mice housed five to a cage (8–12 weeks of age) were used. C57BL/6J mice were used for evoked locomotor rotarod and formalin assays.^35,60,61^ Antinociception was confirmed with the use of CD-1 mice in the CCI nerve assay. The CD-1 strain has been well-validated for antinociceptive^62^ and mechanical anti-allodynic testing.^63,64^ All test compounds were administered using the intraperitoneal (i.p.) route. All animal studies reported herein adhere to ARRIVE guidelines.^65^ Animals were randomly assigned, and researchers were blinded to group treatments. Animals were housed on a 12:12 h light/dark cycle (lights off at 7:00 pm) with *ad libitum* access to food and water except during experimental sessions. All procedures were preapproved by the Institutional Animal Care and Use Committee (University of Florida) and conducted in accordance with the 2011 NIH Guide for the Care and Use of Laboratory Animals.

#### Formalin Test

The efficacy of compound **15** to ameliorate inflammatory nociception was achieved with the use of C57BL/6J mice in the formalin assay as previously described.^53^ After a 10 min pretreatment (i.p.) of vehicle control (saline), CM304 (3–30 mg/kg, i.p.), or compound **15** (3–30 mg/kg, i.p.), an intraplantar (i.pl.) injection of 5% formalin (2.5 μg in 15 μL) was administered into the right hind paw. Time spent licking the right hind paw was recorded in 5 min intervals for 60 min following injection. The last 55 min of assessment was used to determine the inflammatory response stimulus. Data were analyzed as the summed duration of licking hind paw.

#### CCI Assay

CCI was introduced in CD-1 mice that were first anesthetized with isoflurane as described by Hoot et al.^66^ and Cirino et al.^35^ to induce mechanical allodynia.^51−54^ After anesthetization, mice were subjected to surgery where an incision was made along the surface of the biceps femoris of the right hind paw.^66^ Blunt forceps were used to split the muscle and expose the right sciatic nerve. The tips of two 0.1–10 μL pipet tips facing opposite directions were passed under the sciatic nerve to allow for easy passing of two sutures under the nerve, 1 mm apart. The sutures were tied loosely around the nerve and knotted twice, and the skin was closed with 29 mm skin staples. Mice were given a 7 day recovery period prior to the baseline von Frey testing as described below to confirm the induction of hyperalgesia in each mouse. Animals demonstrating allodynia or a response to lower pressure were deemed to have neuropathic pain. Allodynic mice were then administered (i.p.) either vehicle (saline), morphine (10 mg/kg, i.p.), gabapentin (50 mg/kg, i.p.), CM304 (45 mg/kg, i.p.), or compound **15** (10–60 mg/kg, i.p.). Note that gabapentin was tested 1 h postinjection to circumvent known sedative effects that may confound the assay.^67^ Each mouse was then tested for the modulation of tactile allodynia every 20 min up until 80 min post-injection with the use of von Frey testing. The assessment of mechanical allodynia was performed to measure compound **15**’s efficacy against CCI-induced allodynia as described.^51−54^ Mice were habituated on a mesh platform for 1 h prior to testing. Filaments of increasing pressure (0.4–6 g) were applied and then held to the plantar surface of both the injured and uninjured hind paws of mice for approximately 1–2 s prior to drug administration to record baseline responses to a peripheral stimulus. The filaments were applied with increasing strengths, and threshold responses were defined as two hind paw responses per trial of the same filament strength.

Control or test compounds were administered (i.p.), and paw-withdrawal thresholds were again recorded from 20 to 80 min postinjection. Each hind paw was tested in a counterbalanced manner. Each time was measured in triplicate and then averaged. Responsiveness was a clear withdrawal, shaking, or licking of the paw. To account for variability between mice, data are presented as the percent of baseline paw withdrawal thresholds following filament stimulation of the ipsilateral hind paw. The following equation was used: % anti-allodynia = 100 × ([mean paw withdrawal force {g} in control group – paw withdrawal force {g} of each mouse]/mean paw withdrawal force [g] in control group).

#### Rotarod Assay

The rotarod coordination assay was used to assess effects on evoked locomotor activity in C57BL/6J mice administered vehicle (saline, i.p.), morphine (10 mg/kg, i.p.), U50,488 (10 mg/kg, i.p.), CM304 (45 mg/kg, i.p.), or compound **15** (30–60 mg/kg, i.p.) using methods described previously.^68,69^ Seven habituation trials were performed where the last habituation trial was used as an initial baseline of performance. The mice were administered (i.p.) test agents and then evaluated every 10 min in accelerated speed trials (180 s max latency at 0–20 rpm) over a 60 min period. The latency to fall was measured in seconds. Data are reported as the mean percent change from each mouse’s initial baseline latency to fall. Decreased latencies to fall in the rotarod test indicate impaired motor coordination or sedation

#### Statistical Analysis

All data are presented as mean ± SEM. Significance is indicated as *p < 0.05 and was analyzed using two-way ANOVA with Tukey’s post hoc analysis. Statistical analysis was performed with the use of GraphPad Prism 9.0 software. Dose response lines were analyzed by linear or nonlinear regression modeling and ED_50_ values (dose yielding 50% effect) along with 95% confidence limits using each individual data points. The rotarod data are expressed as the % change from baseline performance for each animal’s baseline response.

## Supporting Information Available

Figures of ^1^H and ^13^C NMR spectra of compounds **13**–**16**, **20**–**22**, **23**, and **24** (PDF)
