# Supplementary material for: Development of New Benzylpiperazine Derivatives as σ1 Receptor Ligands with in Vivo Antinociceptive and Anti-Allodynic Effects
Source: ACS Chem Neurosci. 2021 May 21;12(11):2003–12. doi: 10.1021/acschemneuro.1c00106 (PMC8291485; doi:10.1021/acschemneuro.1c00106)
Supplement: Supplementary file 1 — cn1c00106_si_001.pdf [file cn1c00106_si_001.pdf]

Supporting Information

Development of New Benzylpiperazine Derivatives as σ_1 Receptor Ligands with *in Vivo* Antinociceptive and Anti-Allodynic Effects

Giuseppe Romeo,[†] Federica Bonanno,[†] Lisa L. Wilson,[‡] Emanuela Arena,[†] Maria N. Modica,[†] Valeria Pittalà,[†] Loredana Salerno,[†] Orazio Prezzavento,[†] Jay P. McLaughlin,[‡] and Sebastiano Intagliata[*,†]

[†]Department of Drug and Health Sciences, University of Catania, viale A. Doria 6, 95125 Catania, Italy

[‡]Department of Pharmacodynamics, College of Pharmacy, University of Florida, Gainesville, Florida 32610, United States

*Phone: +39-095-738-4053. Email: s.intagliata@unict.it.

Contents:

**Figures S1**–**S18**: ^{1}H NMR and ^{13}C NMR spectra of compounds **13**–**16**, **20**–**22**, **23**, and **24**	p. S2–S10

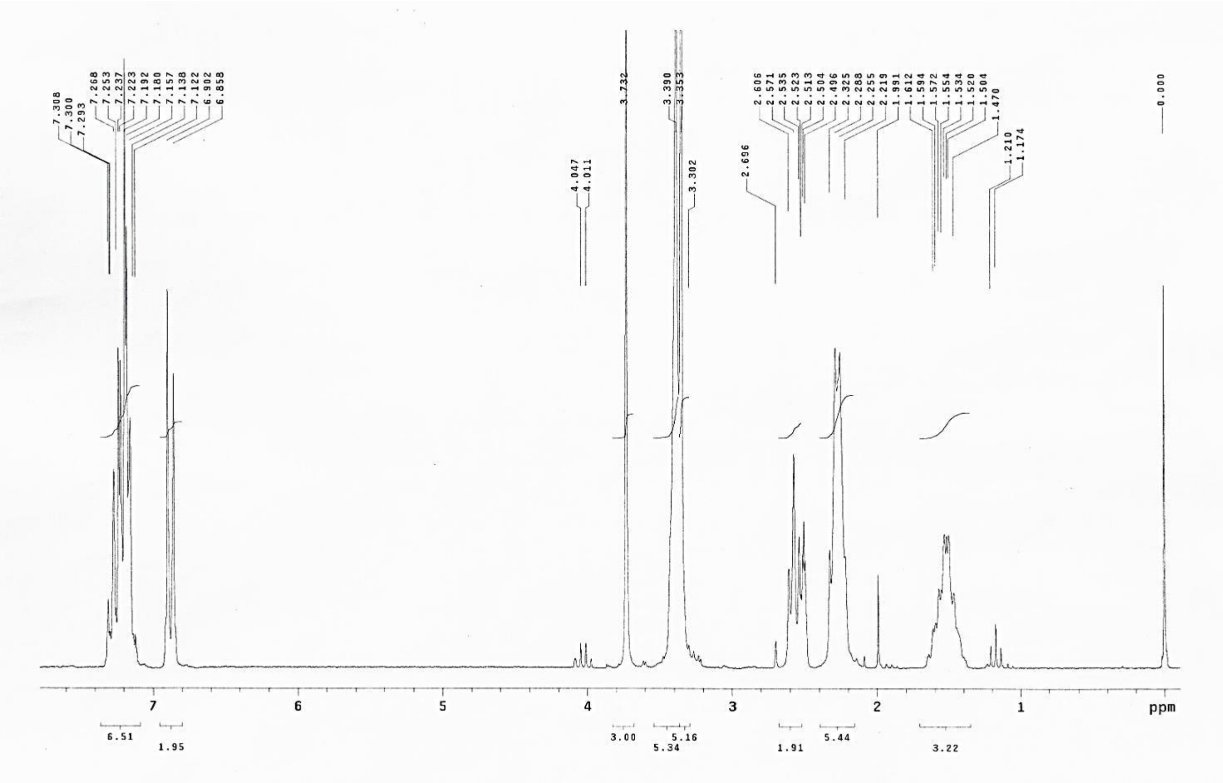

Figure S1. ^{1}H NMR spectrum of compound **13**

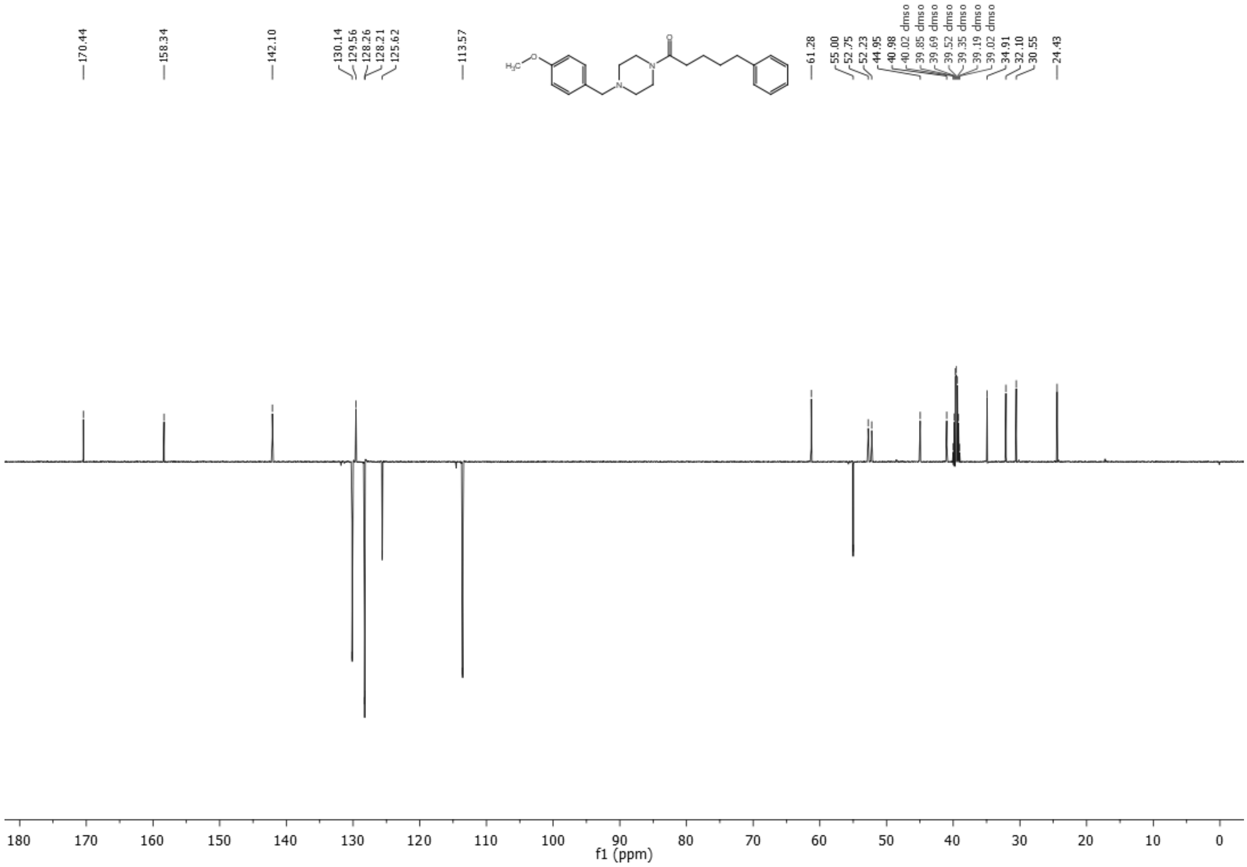

Figure S2. ^{13}C NMR spectrum of compound **13**

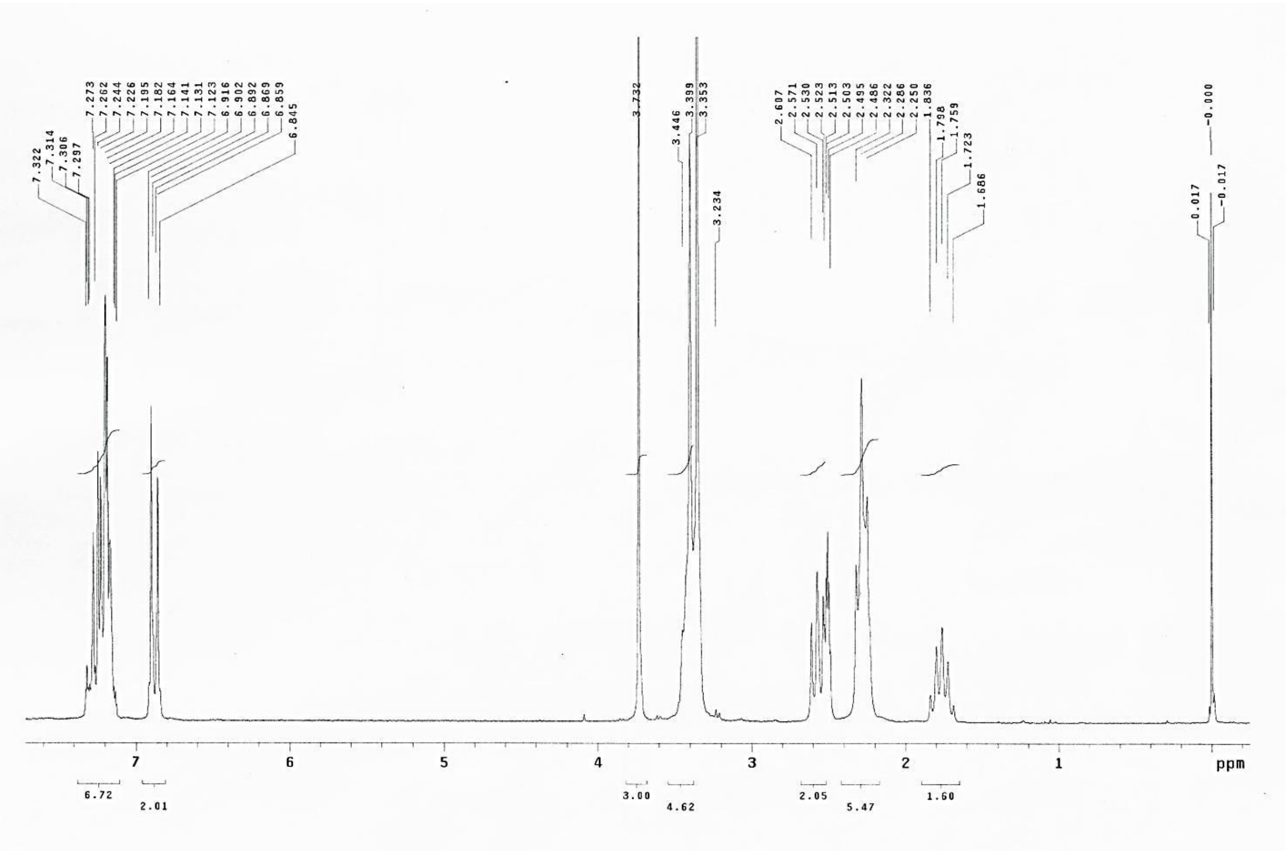

Figure S3. ^{1}H NMR spectrum of compound **14**

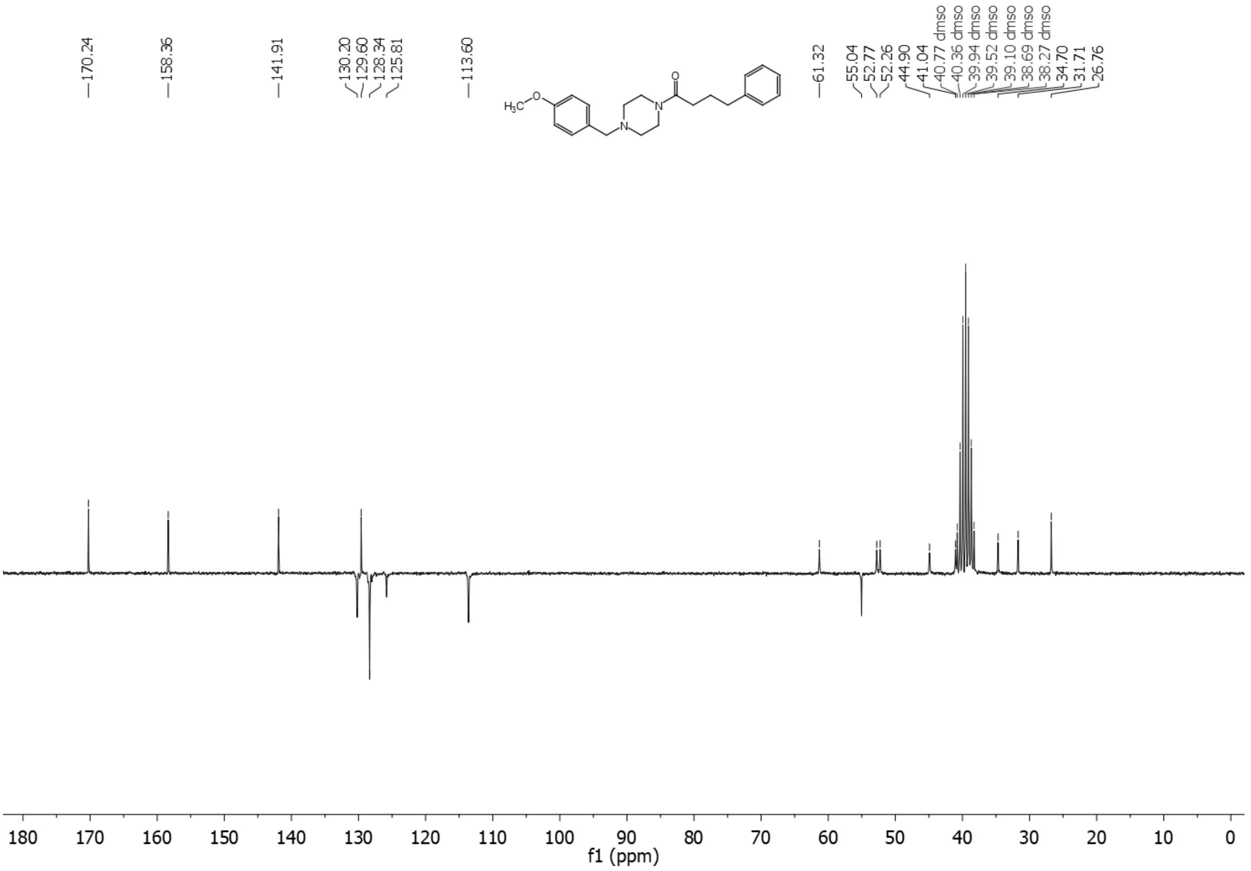

Figure S4. ^{13}C NMR spectrum of compound **14**

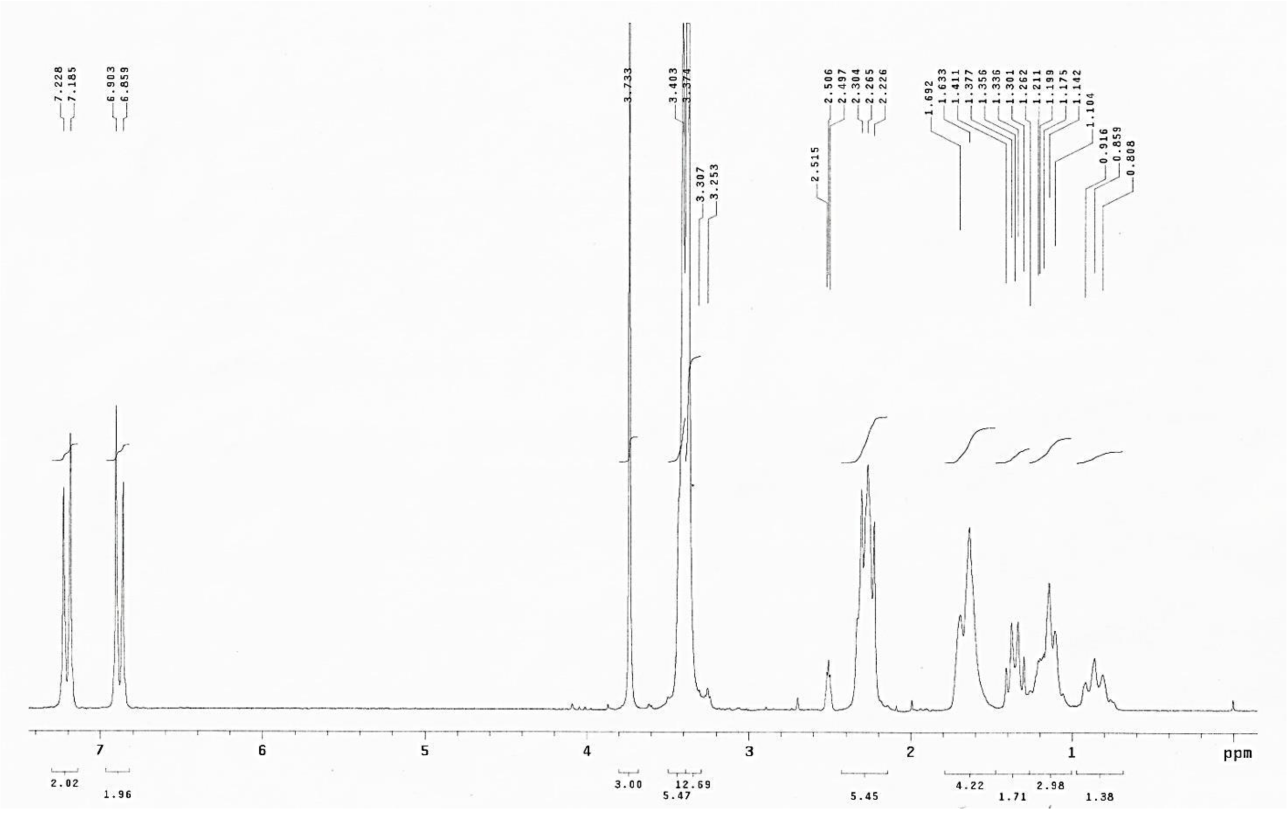

Figure S5. ^{1}H NMR spectrum of compound **15** (free base)

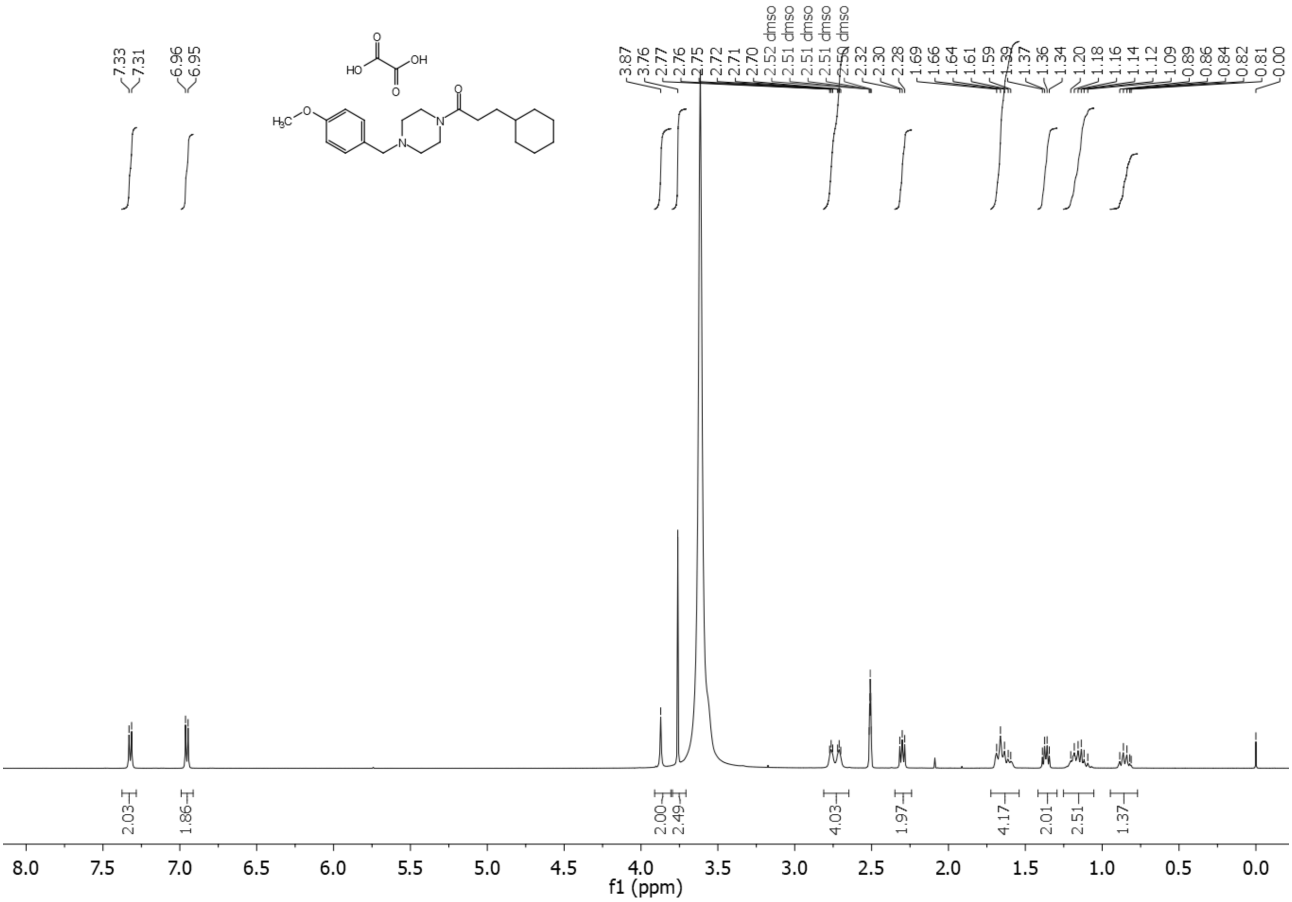

Figure S6. ^{1}H NMR spectrum of compound **15** (oxalate salt)

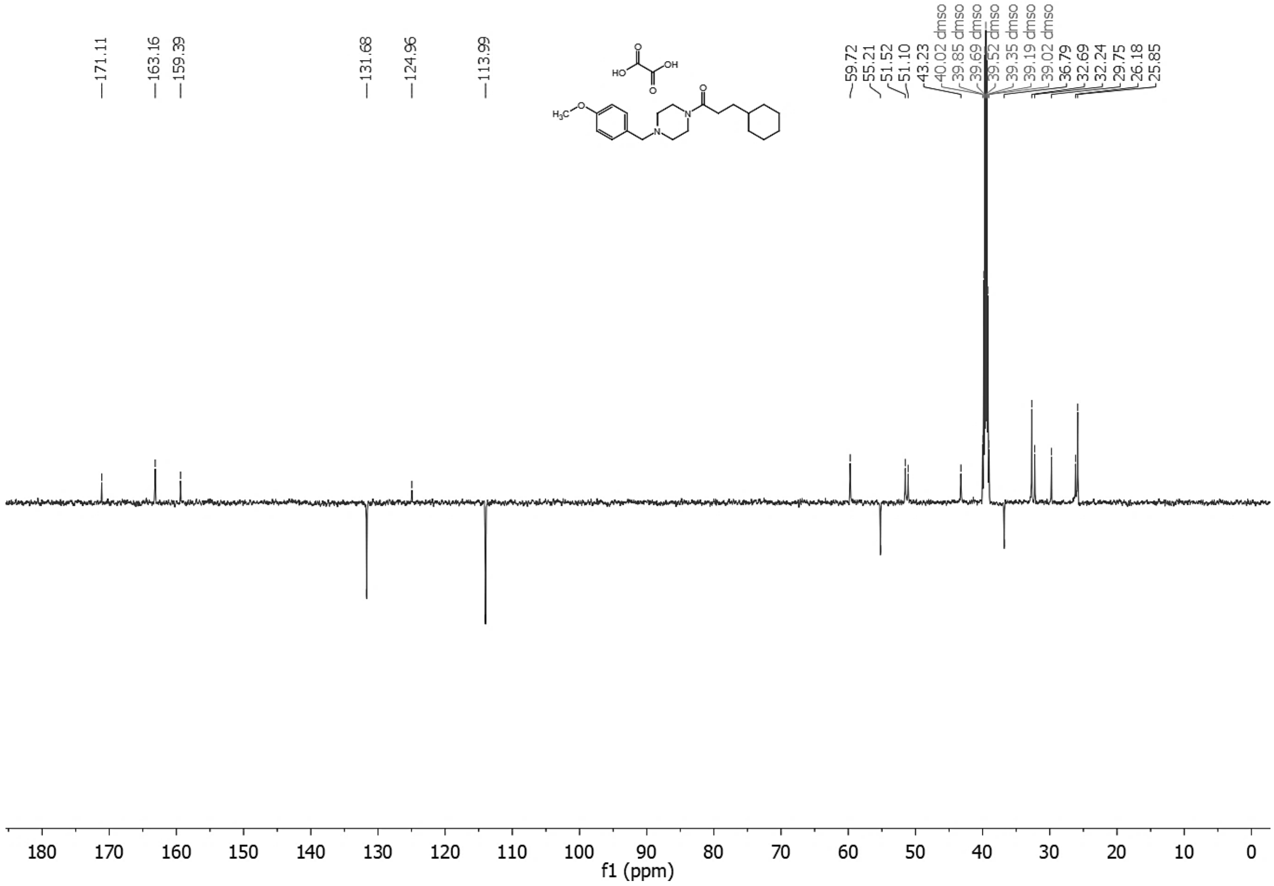

Figure S7. ^{13}C NMR spectrum of compound **15** (oxalate salt).

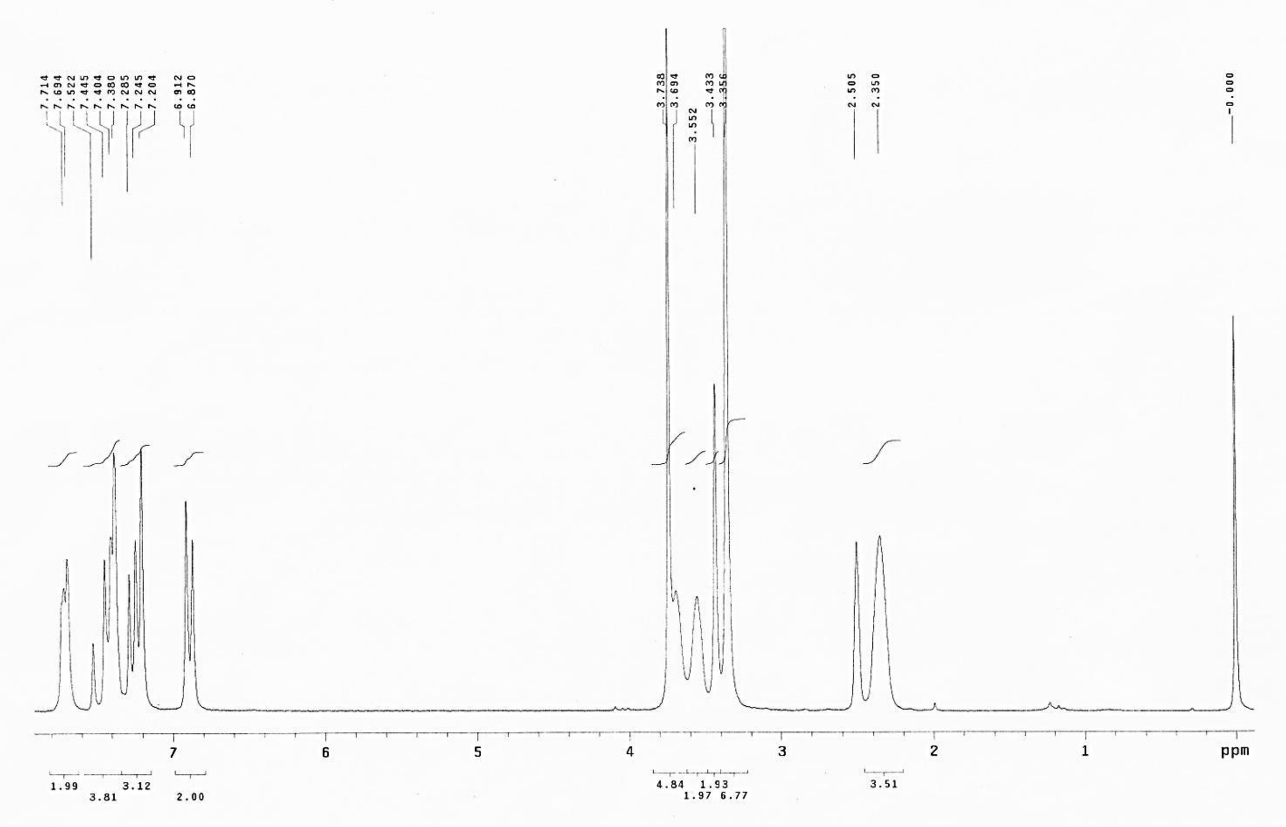

Figure S8. ^{1}H NMR spectrum of compound **16**

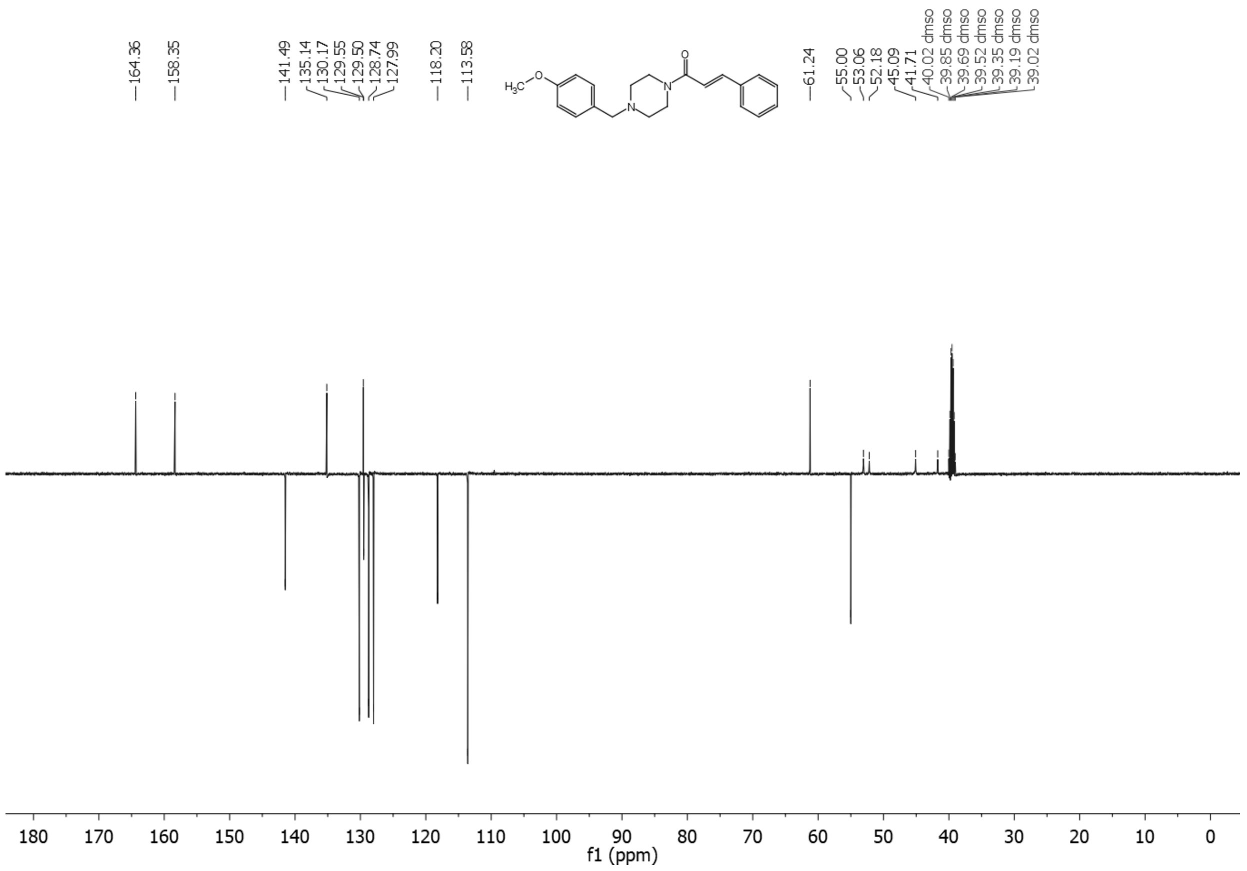

Figure S9. ^{13}C NMR spectrum of compound **16**

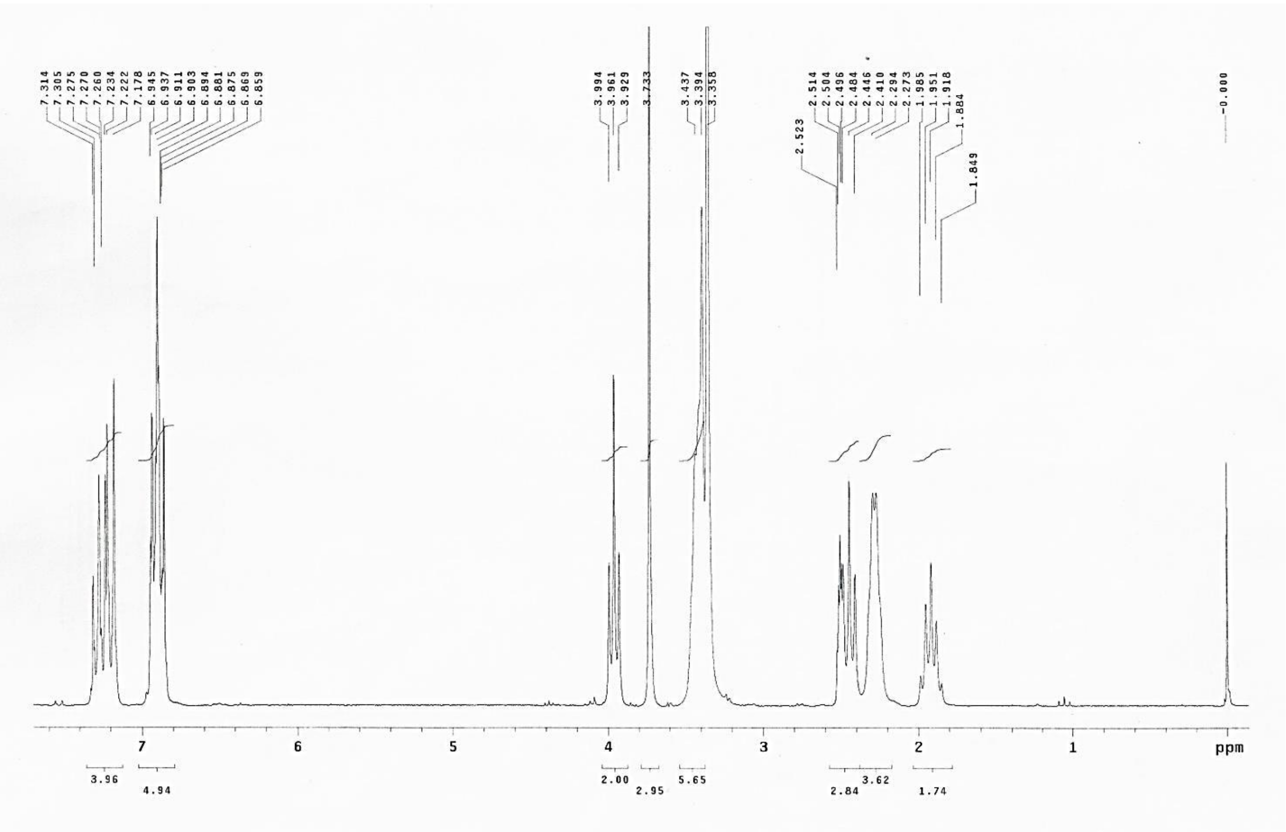

Figure S10. ^{1}H NMR spectrum of compound **20**

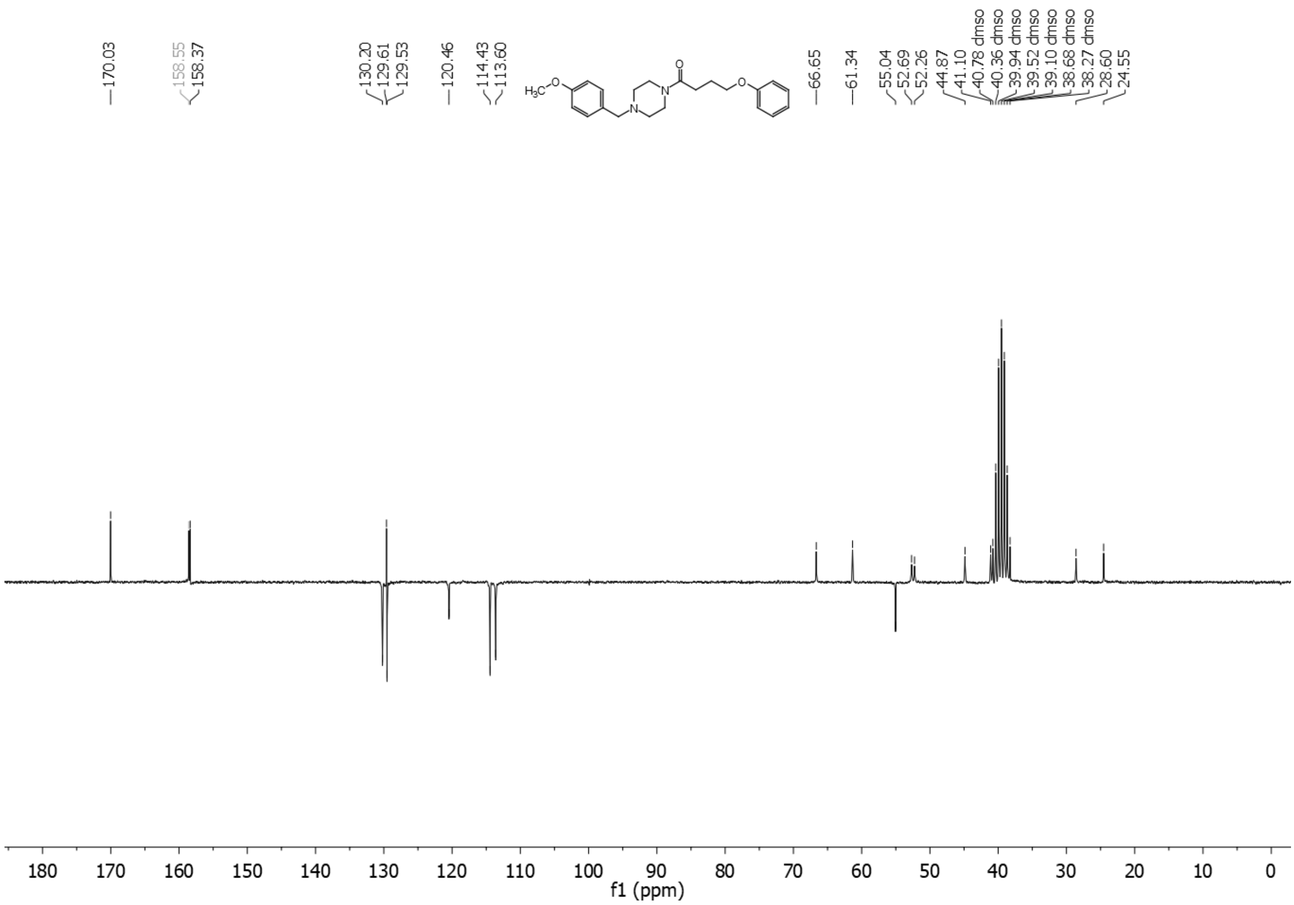

Figure S11. ^{13}C NMR spectrum of compound **20**

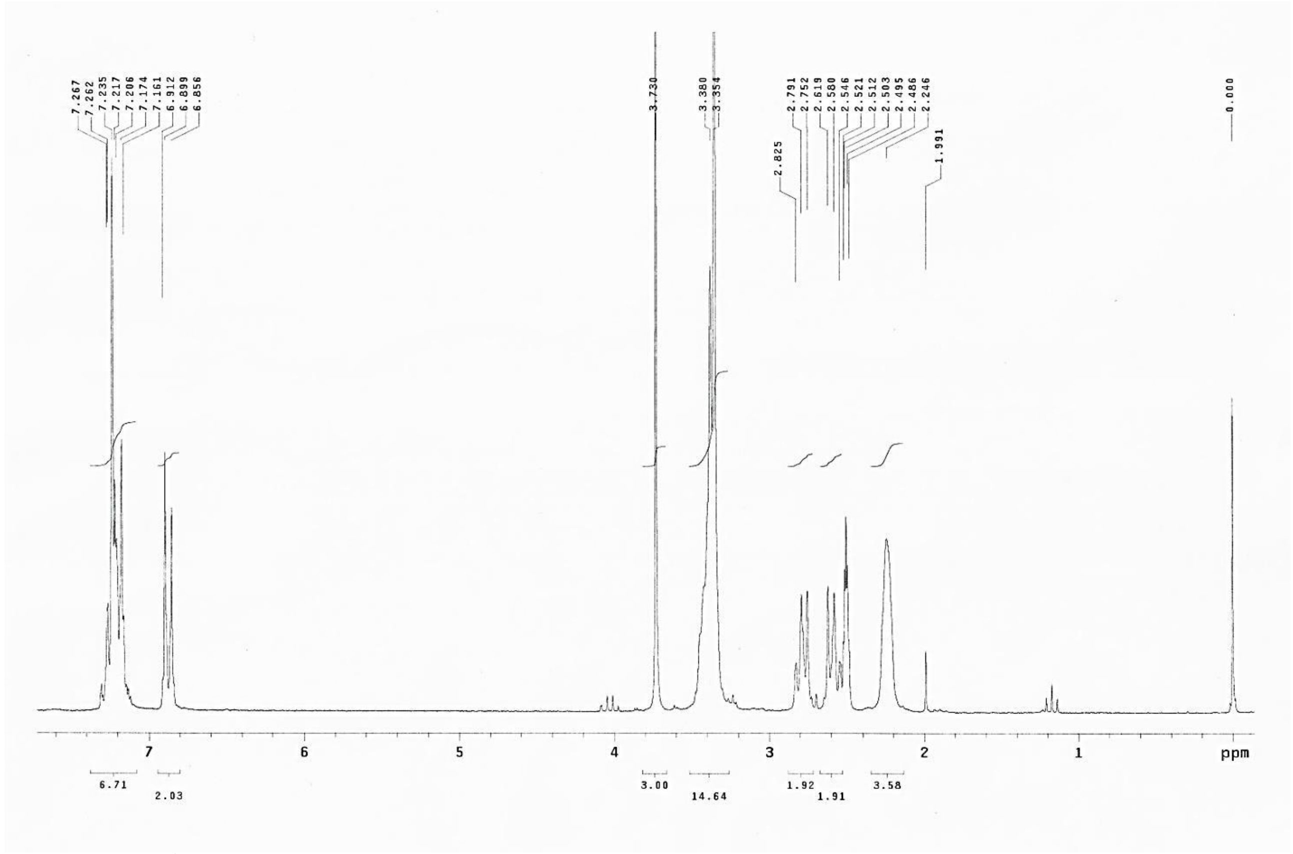

Figure S12. ^{1}H NMR spectrum of compound **21**

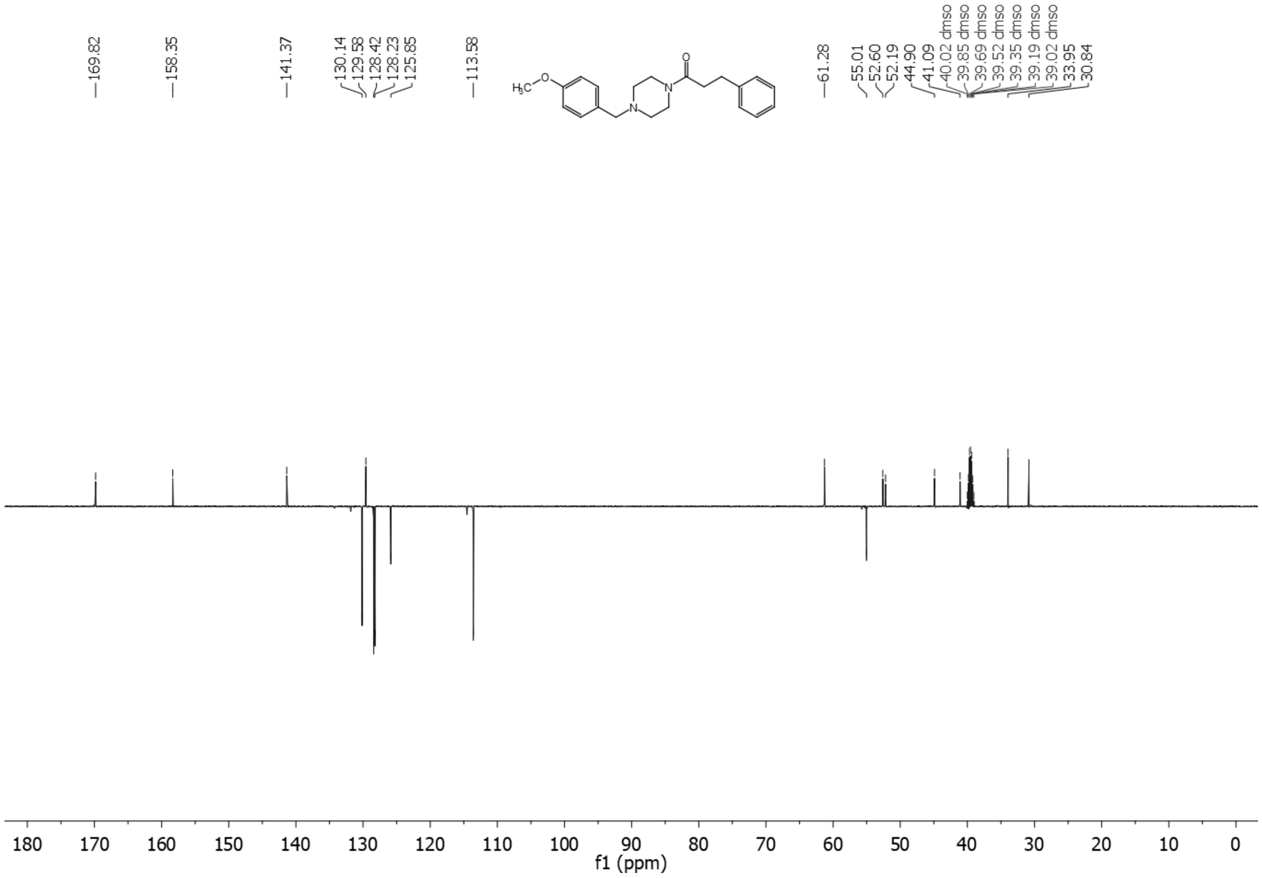

Figure S13. ^{13}C NMR spectrum of compound **21**

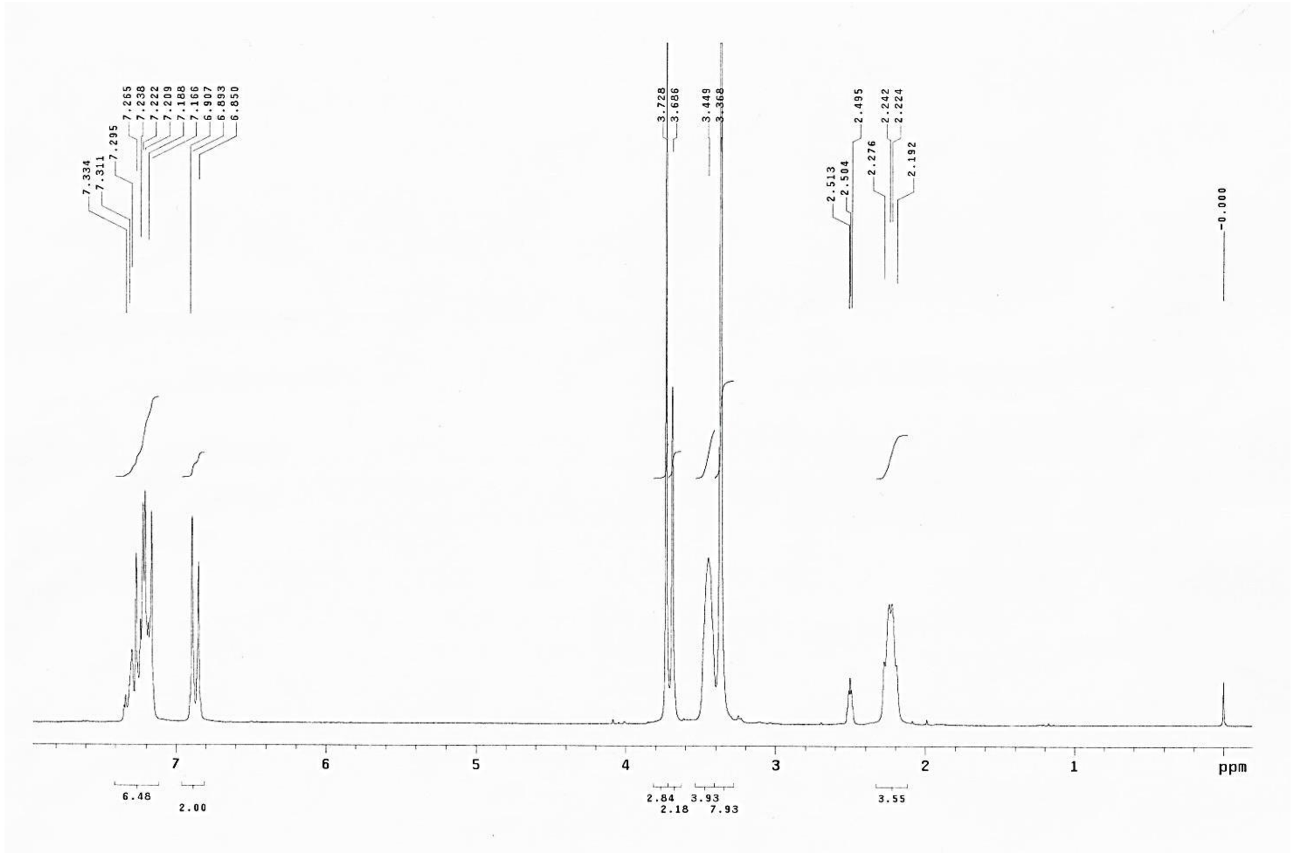

Figure S14. ^{1}H NMR spectrum of compound **22**

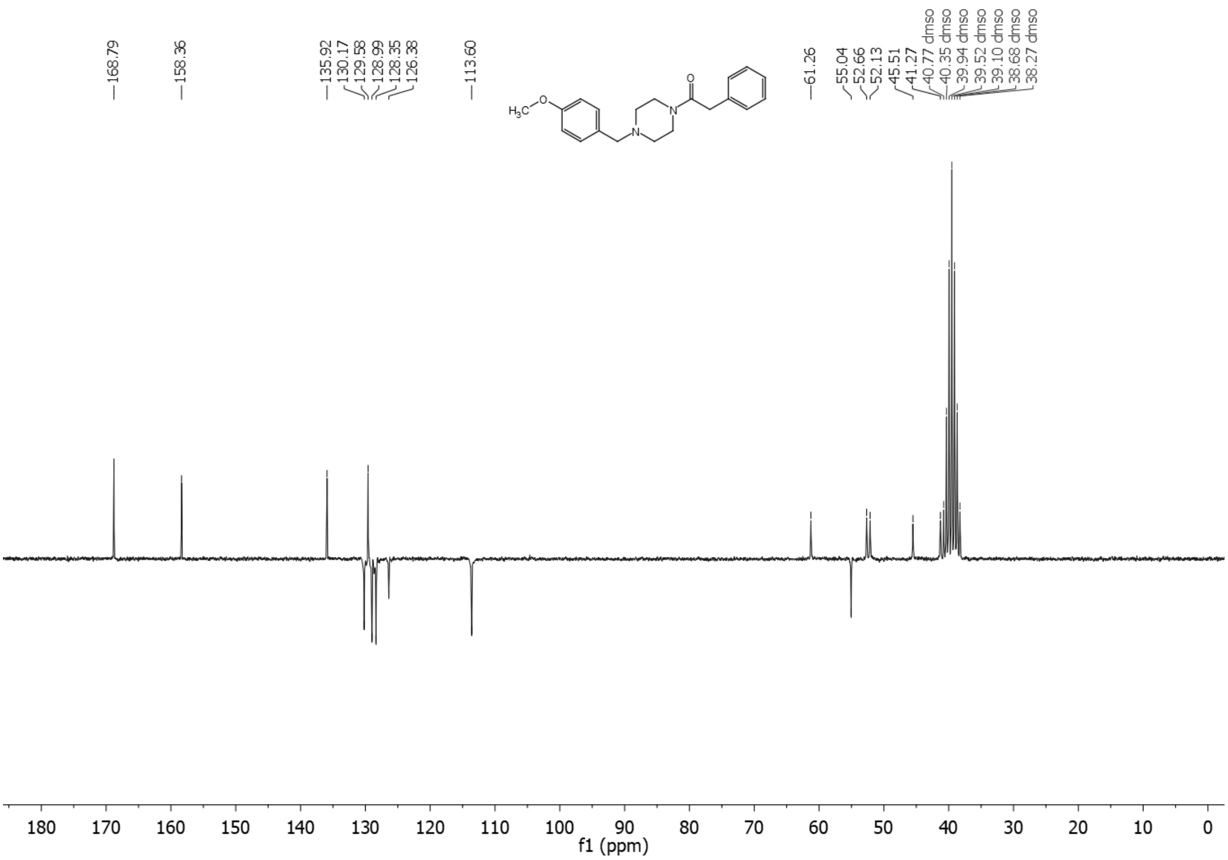

Figure S15. ^{13}C NMR spectrum of compound **22**

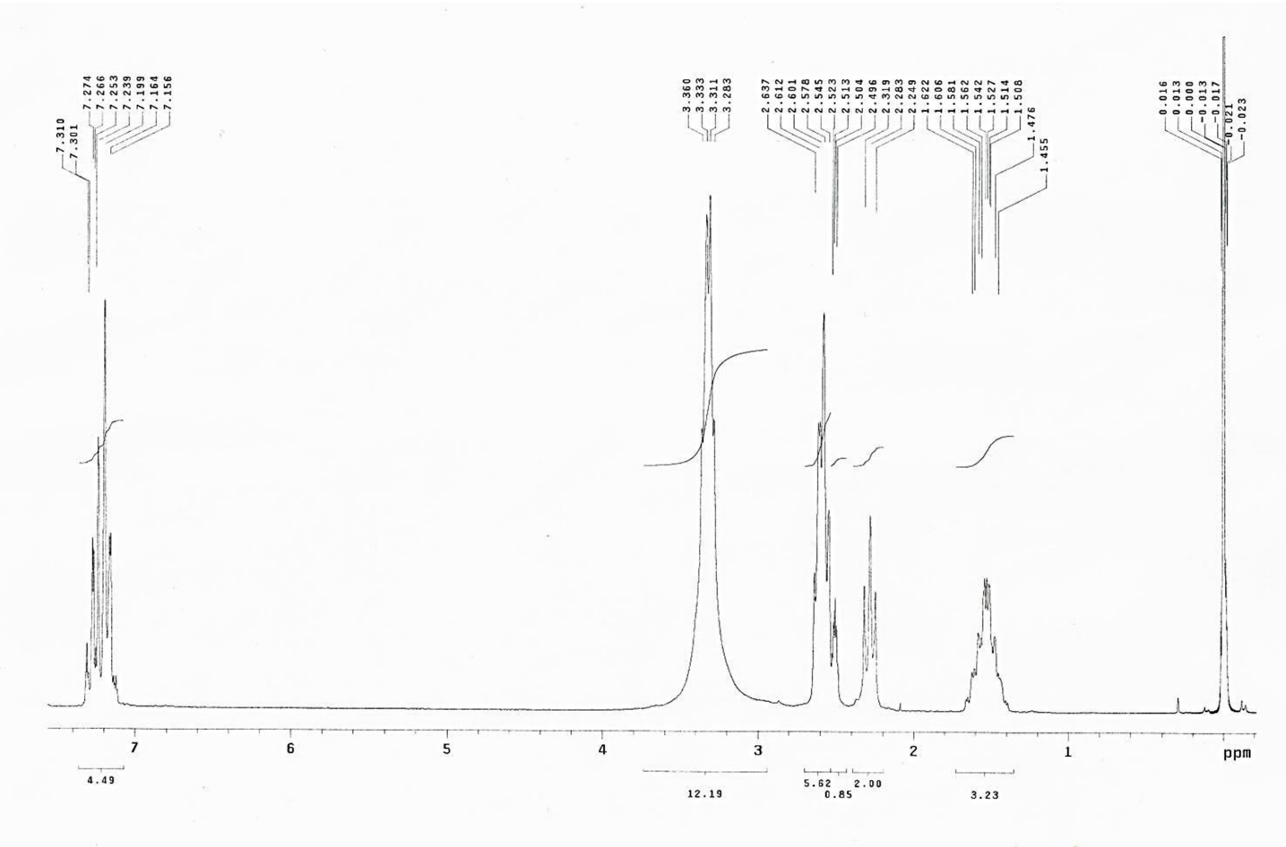

Figure S16. ^{1}H NMR spectrum of compound **23**

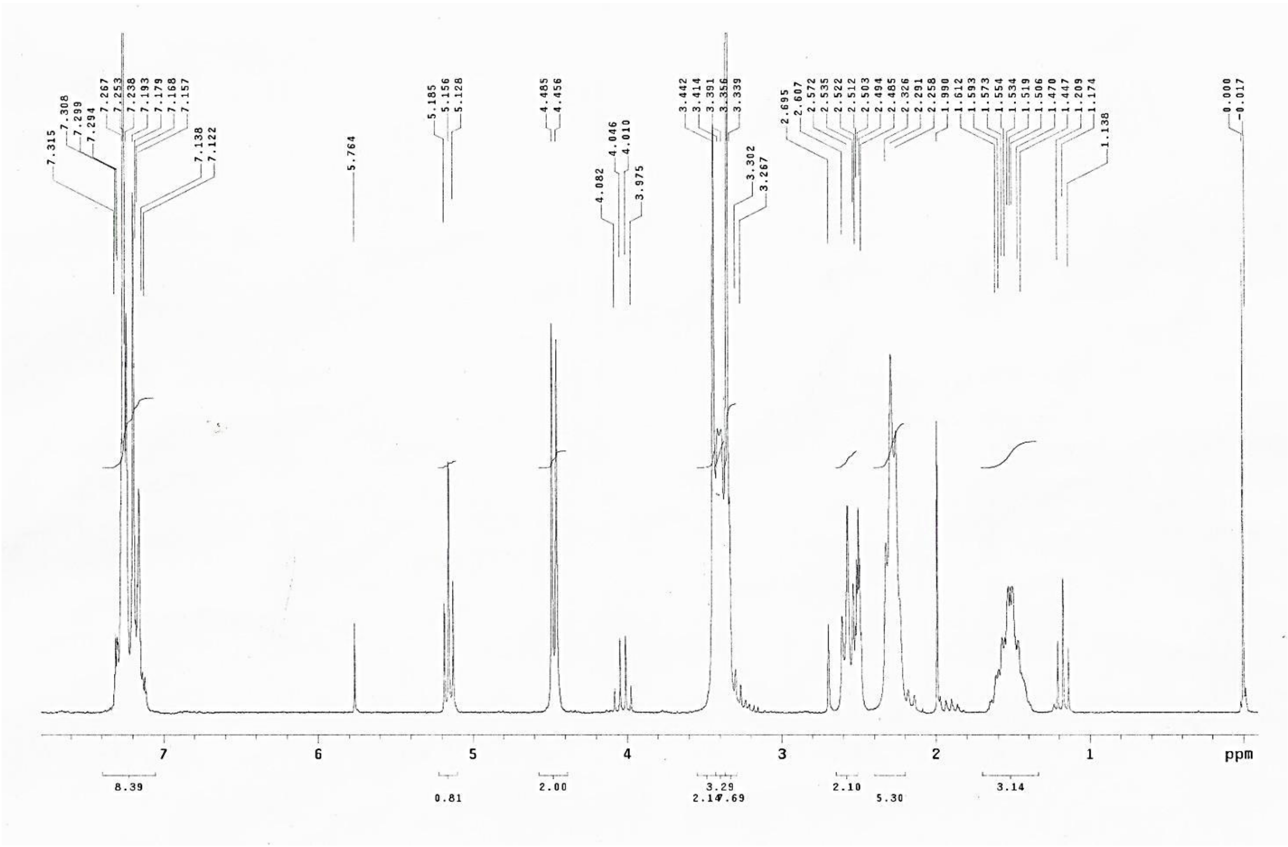

Figure S17. ^{1}H NMR spectrum of compound **24**

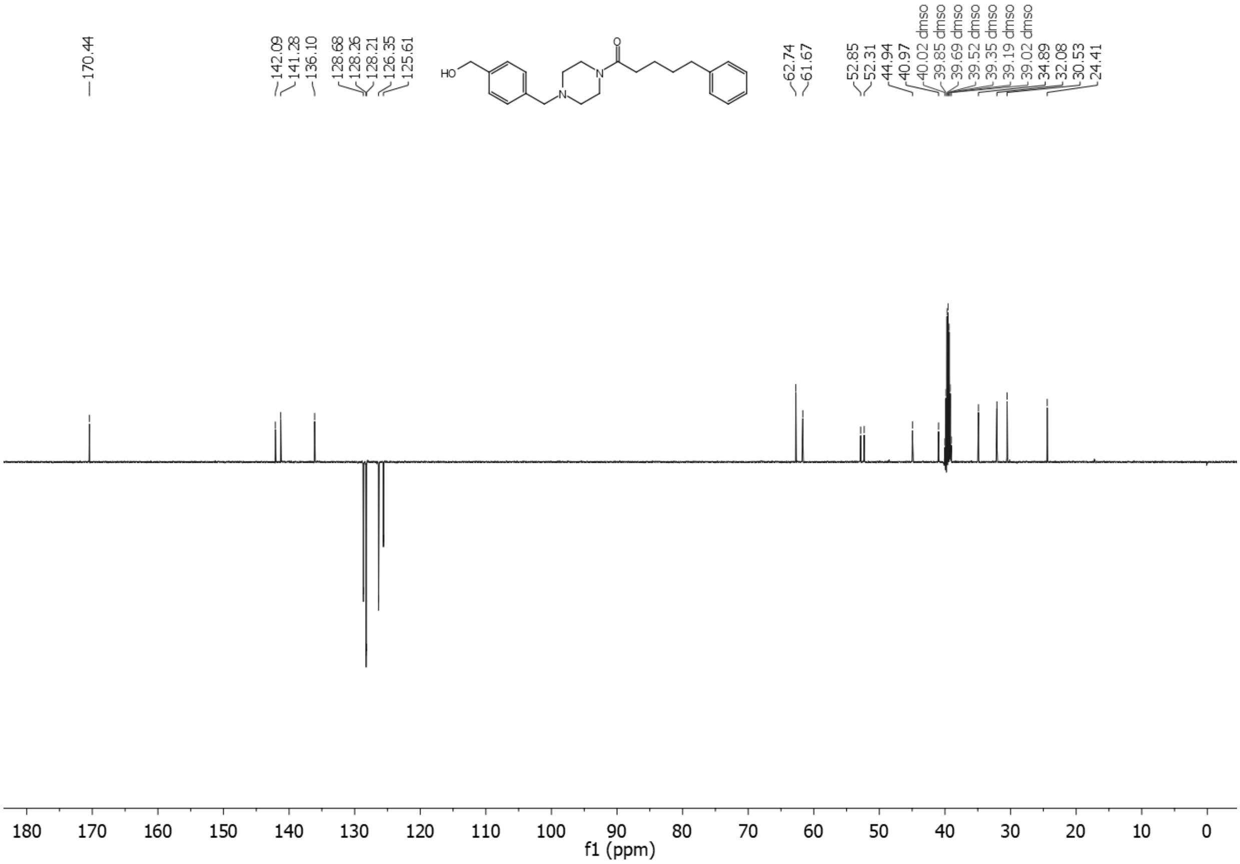

Figure S18. ^{13}C NMR spectrum of compound **24**
